# Enamel formation and growth in non-mammalian cynodonts

**DOI:** 10.1098/rsos.172293

**Published:** 2018-05-16

**Authors:** Rachel N. O'Meara, Wendy Dirks, Agustín G. Martinelli

**Affiliations:** 1 The University Museum of Zoology, Downing Street, Cambridge CB2 3EJ, UK; 2 Department of Anthropology, Durham University, Dawson Building, South Road, Durham DH1 3LE, UK; 3 Departamento de Geociências, Universidade Federal do Rio Grande do Sul, Ave. Bento Gonçalves 9500, 91540-000 Porto Alegre, Rio Grande do Sul, Brazil; 4 Sección Paleontología de Vertebrados, Museo Argentino de Ciencias Naturales ‘Bernardino Rivadavia’, Av. Ángel Gallardo 470, Buenos Aires C1405DJR, Argentina

**Keywords:** enamel increment, enamel development, dental histology, cynodont, mammaliamorph, mammaliaform

## Abstract

The early evolution of mammals is associated with the linked evolutionary origin of diphyodont tooth replacement, rapid juvenile growth and determinate adult growth. However, specific relationships among these characters during non-mammalian cynodont evolution require further exploration. Here, polarized light microscopy revealed incremental lines, resembling daily laminations of extant mammals, in histological sections of enamel in eight non-mammalian cynodont species. In the more basal non-probainognathian group, enamel extends extremely rapidly from cusp to cervix. By contrast, the enamel of mammaliamorphs is gradually accreted, with slow rates of crown extension, more typical of the majority of non-hypsodont crown mammals. These results are consistent with the reduction in dental replacement rate across the non-mammalian cynodont lineage, with greater rates of crown extension required in most non-probainognathians, and slower crown extension rates permitted in mammaliamorphs, which have reduced patterns of dental replacement in comparison with many non-probainognathians. The evolution of mammal-like growth patterns, with faster juvenile growth and more abruptly terminating adult growth, is linked with this reduction in dental replacement rates and may provide an additional explanation for the observed pattern in enamel growth rates. It is possible that the reduction in enamel extension rates in mammaliamorphs reflects an underlying reduction in skeletal growth rates at the time of postcanine formation, due to a more abruptly terminating pattern of adult growth in these more mammal-like, crownward species.

## 1. Introduction

Periodic incremental markings in enamel and dentine represent a permanent record of dental development, produced by the gradual accretion of dental hard tissue in amniote teeth. Different orders of increment differ in temporal periodicity, ranging from sub-daily to annual (reviewed in [1–4]), with rates of formation governed by biological rhythms of differing duration. The use of these incremental markings to infer rates of dental growth and to reconstruct life history and ecology has become well established in extant and extinct mammals [3–15]. While similar studies of incremental markings, particularly using dentine, have been extended to extant and extinct non-mammalian amniotes [16–26], there has been relatively little investigation of the enamel increments of non-mammalian synapsids beyond identification of the presence of periodic markings within the broader context of describing synapsid enamel microstructure [19,23,27–29].

**Figure 1.**
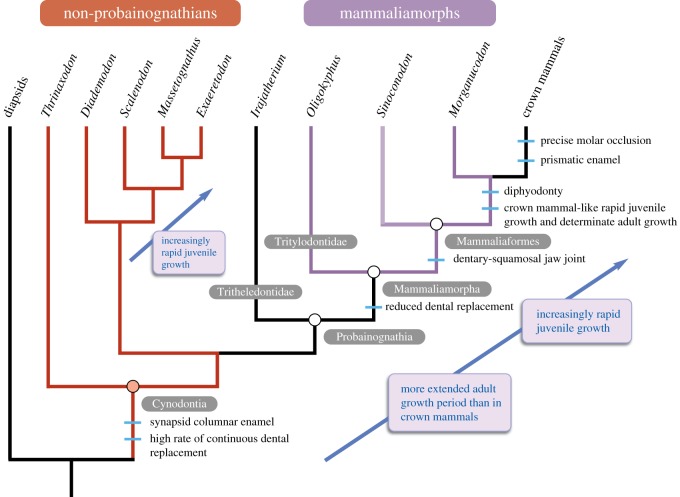
The relationships between non-mammalian cynodonts investigated in this study, and the acquisition of characteristics related to growth and dentition in these animals. Teeth were sectioned from all species with the exception of *Sinoconodon*. In statistical tests, non-mammalian cynodonts were assigned to two different groups, non-probainognathians (red) and mammaliamorphs (purple) and compared with diapsids, non-hypsodont crown mammals and hypsodont mammals. *Irajatherium* was not assigned to any group as its enamel showed a pattern of growth intermediate between non-probainognathians and mammaliamorphs. *Exaeretodon* was excluded from statistical analyses due to its extremely unusual pattern of enamel deposition. Phylogeny after Ruta *et al*. [31].

In this study, for the first time, we compare rates of enamel development inferred from incremental markings in a range of non-mammalian cynodont species. We define Mammalia as a crown group (all descendants of the last common ancestor of monotremes, marsupials and placentals [30]). Mammaliaformes is an apomorphy-based clade designating synapsids with a dentary-squamosal jaw joint; *Morganucodon* is among its most basal known members [30]. Mammaliamorpha is used here *sensu* Rowe [30] as the clade comprising the last common ancestor of Tritylodontidae and crown Mammalia, and its descendants, and thus includes Mammaliaformes. These relationships are illustrated in figure 1, following the phylogeny of Ruta *et al*. [31]. Under these definitions, non-mammalian cynodonts include both non-mammalian mammaliaforms and mammaliamorphs *sensu* Rowe [30].

Improving knowledge of rates of dental growth in non-mammalian cynodonts has the potential to greatly enhance our understanding of a crucial transition in growth patterns and dental characteristics at the base of the mammaliaform lineage. Diphyodonty, the single replacement of antemolars and non-replacement of molars, is a major mammalian apomorphy [32–37] and represents a reduction in the number of tooth replacements from the basal amniote condition of polyphyodonty, or continuous alternating replacement of the dentition throughout life [36,38–40]. The mammaliaform *Morganucodon* is the most anatomically plesiomorphic stem-mammal to have diphyodont tooth replacement ([33,36,37,41–45]; figure 1), and the origin of diphyodonty was probably permitted by the evolution of placental mammal-like skeletal growth patterns in *Morganucodon* [46]. Both rapid juvenile growth and a determinate pattern of adult growth, in which skeletal growth ceases after a maximum size is reached, were probably necessary for the evolution of reduced diphyodont tooth replacement; determinate growth reduces the requirement for larger replacement teeth to fill an expanding jaw [36,47,48], while rapid juvenile growth limits the time period in which teeth of intermediate size are required [32,48].

The origins of diphyodonty and mammal-like skeletal growth patterns are linked with the subsequent evolution of other important mammalian characteristics of the braincase, olfactory cavity and dentition in more crownward mammaliaforms (figure 1). These features include precise molar occlusion [32,47,49], in which it is important to maintain constant relative positions of upper and lower dentitions. This might be compromised by multiple replacements of teeth or more extended periods of jaw growth and also depends on possession of a true dentary condyle articulating with the squamosal. The evolution of diphyodonty and mammal-like growth are also likely to be correlated with the origin (or, more likely, multiple convergent origins [20,50]) of prismatic enamel, which may have evolved to prevent propagation of cracks through enamel [51,52]. Rather than being replaced, the permanent dentition would be required to last throughout the entire adult lifetime of a diphyodont animal. In addition, the evolution of lactation [37,48] and mammalian jaw closing mechanisms [32,47,49,53] are linked with this transition towards more mammal-like growth patterns and dentition. Thus, investigation of growth, and particularly of dental growth, across the cynodont-to-mammaliaform transition has the potential to provide greater understanding of many aspects of the origin of mammalian biology.

Previous studies have investigated growth patterns in non-mammalian cynodonts during this crucial transition, chiefly using bone histology methods (e.g. [54–59]), assessment of ontogenetic series (e.g. [60,61]) or comparison of size ranges of specimens (e.g. [36,37,46,62]). In this study, we further explore the link between growth patterns and dentition by investigating absolute rates of growth within the dentition itself, a method not previously applied to these animals. We investigate enamel deposition and extension rates in *Morganucodon*, the most plesiomorphic known diphyodont mammaliaform, in more stemward cynodonts, and in crown mammals and diapsids, in order to assess at what point in the cynodont lineage mammal-like patterns of enamel growth become evident.

### 1.1. Enamel microstructure in mammals and non-mammalian cynodonts

The development of mammalian enamel begins with the differentiation of ameloblasts over the dentine horns, located under the future cusps of the tooth. Differentiating ameloblasts extend towards the cervix (the constricted junction between the crown and the root) along the enamel–dentine junction (EDJ), increasing the height of the tooth crown [63]. Ameloblast and odontoblast differentiation is reciprocally induced (each cell type must receive signals from the other in order to differentiate), and so, dentine-secreting odontoblasts differentiate almost simultaneously along the outer dentine surface of the EDJ [64,65]. During enamel development, ameloblasts are displaced progressively further from the EDJ towards the future outer enamel surface, through the secretion of enamel matrix. Initially, newly secreted enamel contains enamel specific proteins, which regulate the growth of enamel apatite crystallites. Expansion of these crystallites and removal of organic matrix then follow during the maturation phase [66,67], although the precise role of the matrix protein amelogenin has recently been questioned [68].

**Figure 2.**
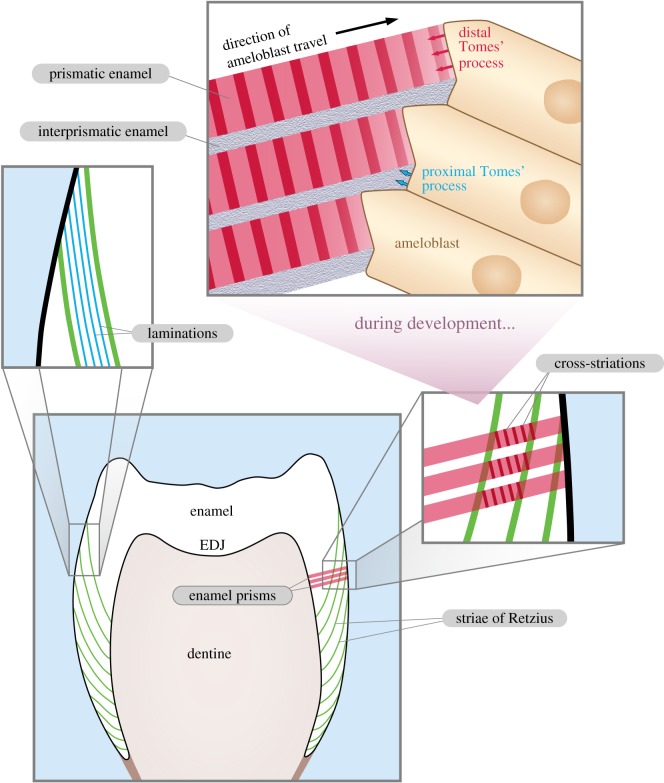
The periodic increments of mammalian enamel and their development. Striae of Retzius (green) represent the forming front of enamel as it is deposited from cusp to cervix along the EDJ. Cross-striations and laminations both have a daily periodicity, but laminations follow a course parallel to the striae of Retzius, while cross-striations are formed perpendicular to enamel prisms, and thus may be oblique to striae of Retzius. Enamel is secreted by ameloblasts moving progressively further from the EDJ. Prismatic enamel is produced by the distal Tomes' process (red), and interprismatic enamel by more proximal portions of the Tomes' process (blue). In extant mammals, the distal Tomes' process is initially absent during early enamel development and also regresses towards the end of development. Thus, during these stages of development, enamel is produced across a relatively flat, ‘proximal', secretory surface, forming regions of interprismatic enamel close to the EDJ and outer enamel surface. Laminations are most frequently observed in these interprismatic regions in extant mammals, forming in line with the flat secretory surface of the ameloblasts. Lower frames of figure follow Smith [3].

A fundamental structural unit of mammalian enamel is the enamel prism. Prisms extend without splitting or merging from close to the EDJ to close to the outer enamel surface and are formed from bundles of long ribbon-like carbonato-apatite crystallites with long axes running in the same longitudinal direction as the prism [69–72]. Each prism head is produced by a single ameloblast and therefore traces the path of that ameloblast from the EDJ to the outer surface (figure 2). Adjacent prisms may decussate, or cross each other's paths, strengthening the enamel structure [70,73]. Between enamel prisms is interprismatic enamel (figure 2, top panel), in which parallel crystallites are oriented obliquely from the crystallites of the prisms.

The structure which secretes enamel is a projection of the ameloblast known as the Tomes' process. Initially, during enamel secretion, an ameloblast has a relatively flat secretory surface. This is known as the proximal portion of the Tomes' process, and it secretes only prismless enamel, with crystallites orientated in the same direction as interprismatic enamel [74,75]. The ameloblast later develops an extended distal portion of the Tomes' process, as an outgrowth of the proximal portion (figure 2, top panel). This has an associated enamel secretory site distinct from the proximal secretory sites that are now located around the base of the extended Tomes' process [15,72]. The proximal site secretes interprismatic enamel to form an encircling pit into which the distal site secretes the prismatic enamel. The distal portion of the Tomes' process regresses towards the end of enamel secretion, and a layer of prismless enamel is formed close to the outer enamel surface [15,72]. Thus, thin layers of prismless interprismatic enamel are often observed close to the EDJ and the outer enamel surface, with prismatic enamel between them.

The enamel of non-mammalian cynodonts differs from that of mammals as it lacks the distinction between prismatic and interprismatic enamel. An exception to this is the tritheledontid *Pachygenelus* which is reported to have a plesiomorphic prismatic enamel structure [27,50]. A variety of terms have been applied to the enamel types observed in non-mammalian cynodonts and in diapsids including ‘aprismatic’, ‘non-prismatic’, ‘pseudo-prismatic’ and ‘preprismatic’ (reviewed in Sander [19]). To clarify the terminology of prismatic structure in non-mammals, Sander [19] suggests that all enamel lacking prismatic structure be referred to as ‘prismless’, and this is the term we use throughout this study.

The prismless enamel of the majority of non-mammaliaform cynodonts, and also of *Morganucodon*, is of a type identified as synapsid columnar enamel ([20,76,77]; figure 1) in which the enamel is arranged in parallel, non-decussating columns perpendicular to the outer enamel surface [29,71,76]. This resembles the columnar enamel of many reptiles [19,70]. These columns are elongate, with some extending almost to the outer enamel surface, although others are discontinuous [71]. The ameloblasts of cynodonts are likely to have followed a similar path to those of mammals, from the EDJ to the outer enamel surface. However, hypotheses of amelogenesis in synapsids suggest that the mineralization front lagged behind the secretion front during maturation of the enamel, so that there is not necessarily a one-to-one relationship between individual ameloblasts and columnar units [71]. It is also likely that ameloblasts secreted enamel across a flat secretory surface and lacked Tomes' processes with separate sites for prismatic and interprismatic enamel [71].

### 1.2. Periodic increments in mammalian enamel

Several different types of enamel increment, each with a consistent periodic repeat interval, have been identified among extant mammals. These are illustrated in figure 2. Increments form as the rhythmic changes in ameloblast secretion over different time scales alter the mineral composition of enamel [78,79]. These can be divided into three basic types, sub-daily or intradian, daily or short period and supra-daily or long period. In primates, the daily increments in enamel are cross-striations [1,6], while in artiodactyls and perissodactyls, the daily increments are laminations [15,80–83]. The supra-daily lines are referred to as striae of Retzius or Retzius lines [4]. There have not been enough studies of other mammals to determine the periodicity of the various lines representing incremental growth, although they have been identified in a wide range of mammals (e.g. [9,12,84,85]). In primates, daily cross-striations and striae of Retzius correspond with short- and long-period lines in dentine, known as lines of von Ebner and Andreson, respectively [86–88].

Studies using vital labelling of forming enamel have demonstrated that cross-striations have a 24 h periodicity in primates [3,89,90], while laminations have been demonstrated to have a daily periodicity in artiodactyls [14,15,80]. Under polarized light microscopy, cross-striations appear as pairs of light and dark bands crossing enamel prisms perpendicularly, parallel to the secretory face of the Tomes' process ([78]; see figure 2, top panel). Laminations, despite their daily nature, differ from cross-striations in that they do not necessarily cross enamel prisms perpendicularly, but rather follow a course parallel to the striae of Retzius [3,14,15,80–82,91]. The daily nature of cross-striations is often used to infer the rate and duration of formation of other enamel increments in both primates and other mammals, in which they have also been assumed to be daily. Sub-daily lines (i.e. under 24 h) have also been observed between cross-striations in primate enamel; they may have a 12 h rhythm, although their appearance can be variable and localized [3]. Alternatively, what appear to be sub-daily lines in enamel with daily laminations may represent the same circadian biorhythm expressed in the interprismatic enamel, offset by the distance between the proximal and distal ends of the Tomes' process [15].

Striae of Retzius (figure 2) are longer period features than cross-striations and represent successive positions of the forming front of enamel [3,78]. They originate from the lateral or shoulder region of the ameloblast [3,92], and so, cross the enamel prisms at an oblique angle (in contrast to the perpendicular cross-striations). Striae of Retzius form successive dome shapes over the dentine horn and extend towards the cervix as enamel is gradually accreted. In cuspal enamel, the striae do not reach the outer enamel surface, but in lateral enamel they manifest as perikymata (shallow grooves) on the enamel surface [5,78,93].

The number of cross-striations between adjacent pairs of striae of Retzius is known as the repeat interval. It is consistent within an individual [94,95] in primates, but varies between, and occasionally within, species [95,96]. Similarly, multiple laminations may be present between adjacent striae of Retzius [81,84]. It is possible for the repeat interval of cross-striations or laminations to be one, in which case striae of Retzius can themselves represent daily intervals [10,14,15,80]. The biological mechanism underlying the formation of striae of Retzius has not been fully established. They may reflect the interaction of two different shorter period rhythms [97,98], although this does not account for all variation in striae of Retzius repeat intervals observed in extant primates [3]. Alternatively, they may represent periodic intensification of the processes underlying cross-striation formation [99]. A recent hypothesis suggests that they are the expression of a biorhythm underlying growth and bone formation in mammals, mediated by the hypothalamus through a neuroendocrine mechanism [9].

The mechanism by which laminations are produced is debated. Tafforeau *et al*. [81] suggest that they represent three-dimensional alignments of cross-striations, while Smith [3] argues that laminations and cross-striations are morphologically distinct, with laminations formed across the entire secreting front of enamel. In the latter hypothesis, it is chiefly the interprismatic growth regions which contribute to lamination formation, in contrast to the process of prism cross-striation formation for which the prismatic growth regions are responsible. This view of a separate origin of laminations from cross-striations is supported by Kierdorf *et al*. [14], who find that lamination formation in the largely interprismatic inner enamel of sheep is associated with the interprismatic growth region of the proximal Tomes' process, rather than the prismatic enamel growth region of the distal Tomes' process (figure 2, top panel). Kierdorf *et al*. [15] also suggest that a similar explanation for lamination formation holds in those regions of dwarf domesticated suid enamel where interprismatic enamel dominates. However, in those regions where prismatic rather than interprismatic enamel dominates, but where laminations are visible, alignment of cross-striations, as described by Tafforeau *et al*. [81], may best explain the appearance of laminations. Thus, the two major explanations for lamination formation are not necessarily incompatible. Note that, regardless of differing interpretations of the mechanism behind lamination formation, all the above authors agree that laminations are daily in nature, and that laminations can be used to calculate rates of enamel formation and crown formation times [3,14,15,81].

### 1.3. Periodic increments in non-mammalian cynodont enamel

Periodic increments have previously been reported in the enamel of several species of non-mammalian synapsid. Grine *et al*. [27] found lines running parallel to the enamel surface in both *Diademodon* and *Thrinaxodon*, and identified these bands as homologous with mammalian striae of Retzius. Incremental lines identified as striae of Retzius have also been reported in the enamel of *Tritylodon* [23,100]. Similar bands have been observed in the tritylodontid *Dinnebitodon* ([76]: appendix), *Massetognathus* (fig. 4 in [28]) and *Exaeretodon* [29].

In this study, we further investigate the incidence of such lines in a taxonomically broad range of non-mammalian cynodonts, and use them to calculate rates of enamel secretion and extension of the crown. We assess how dental growth rates changed throughout the non-mammalian cynodont lineage, and how these changes may be linked with the evolution of mammal-like skeletal growth patterns and reduced dental replacement, characteristics which are a hallmark of the cynodont-to-mammaliaform transition.

## 2. Material and methods

### 2.1. Specimens

We sectioned teeth from both extinct non-mammalian cynodonts and from extinct and extant crown crocodilians and mammals. The non-mammalian cynodont species investigated were *Thrinaxodon liorhinus*, *Diademodon* sp., *Scalenodon angustifrons*, *Massetognathus pascuali*, *Exaeretodon riograndensis*, *Irajatherium hernandezi*, *Oligokyphus* sp. and *Morganucodon watsoni*. Table 1 details the species and tooth types sectioned. To compare dental growth rates between species, we sectioned teeth from similar loci (posterior lower postcanines) where possible, although the difficulty of obtaining rare fossil specimens meant that in some cases, we had to use teeth from upper postcanine loci (table 1). In *Morganucodon* and all extant mammals, almost all specimens sectioned were lower second or third molars. The lack of differentiation between premolars and molars in non-mammaliaform cynodonts made it difficult to section teeth directly homologous with m2 or m3. Therefore, in non-mammaliaform cynodonts, lower postcanines from the middle or posterior of the tooth row were sectioned for most species (see table 1 for exceptions). All the extant mammal and *Morganucodon* teeth sectioned were from the permanent dentition, and in all other species, the teeth sectioned were from adult-sized individuals.

**Table 1.** Descriptions of specimens sectioned. UMZC, University Museum of Zoology Cambridge; CRILAR, Centro Regional de Investigaciones Científicas y Transferencia Tecnológica, Anillaco, La Rioja, Argentina; UFRGS, Universidade Federal Rio Grande do Sul, Porto Alegre, Brazil. WMU and HT accessioned specimens can be accessed at the School of Dental Sciences, Newcastle University.

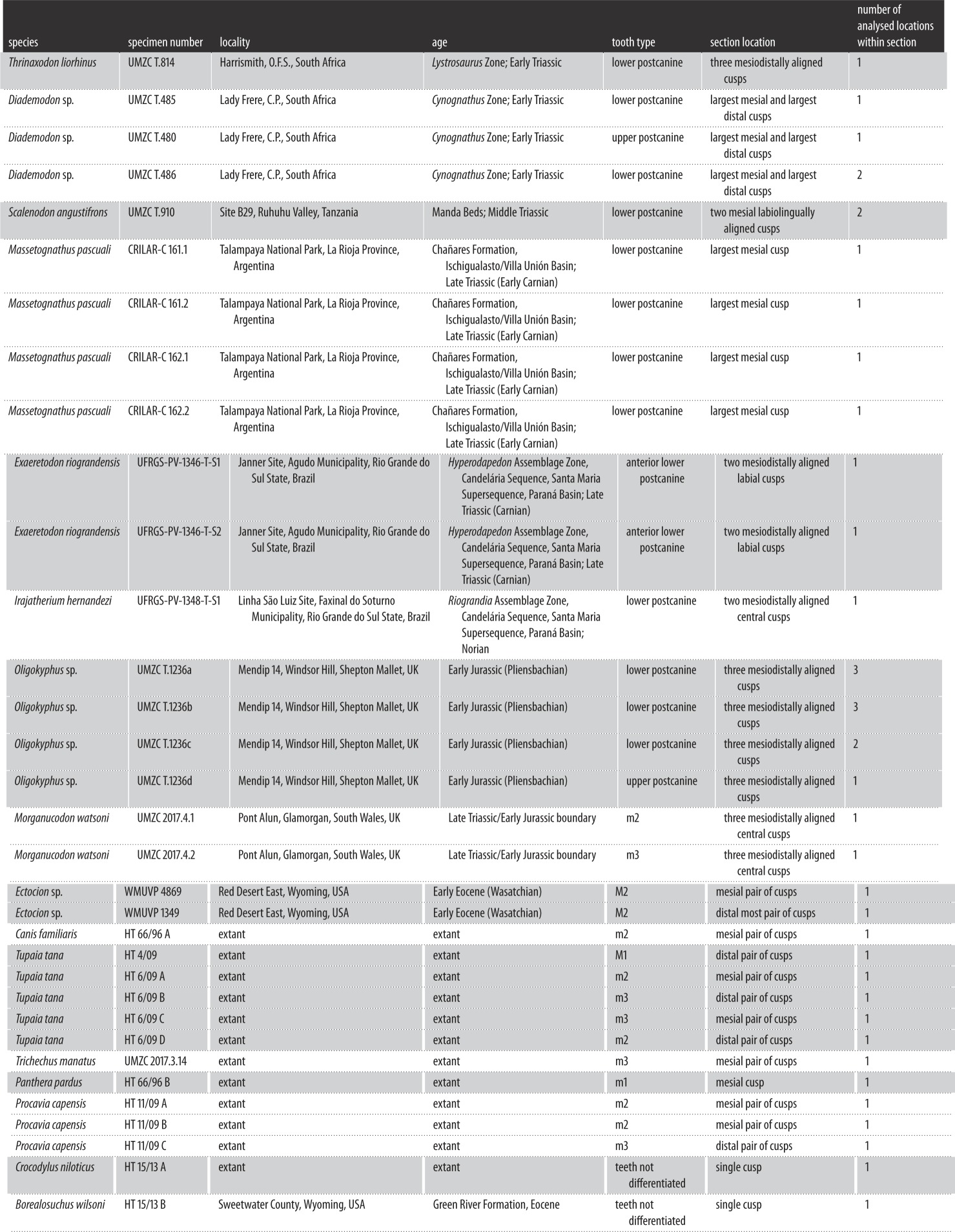

### 2.2. Histological sectioning

Histological section preparation broadly followed the methods detailed in Reid *et al*. [101]. The plane of section is important in the preparation of histological tooth sections; only by sectioning through both the first and last formed enamel can the entire chronology of enamel formation be captured in a single section. This requires the section to pass precisely through the midline of a cusp, in the longitudinal plane, in order to ensure that the earliest formed enamel at the tip of the dentine horn [3,102] is not obscured by later-formed enamel in an obliquely cut section.

Teeth were sectioned longitudinally, from crown to cervix through the principal cusps in the postcanines of all non-mammalian cynodonts. Varied tooth morphologies make it difficult to homologize cusps across cynodont genera. Therefore, because it was not possible to take sections through homologous locations in the teeth of these animals, sections were taken through the largest cusp of each species. This maximizes the length of EDJ, which can be observed in a section, and, if the entire length is well preserved in fossil specimens, allows a maximum crown formation time to be calculated. Where the morphology of the tooth permitted, multiple cusps were included in a section (table 1).

In addition, sections were taken through the principal cusps of teeth of two diapsid species (the extant *Crocodylus niloticus* and an extinct Eocene crocodilian, *Borealosuchus wilsoni*) and six crown mammal species (*Canis familiaris*, *Tupaia tana*, *Panthera pardus*, *Procavia capensis*, *Trichechus manatus* and the extinct phenacodontid *Ectocion* sp.; table 1).

Specimens marked for sectioning were transferred through graded concentrations of alcohol into acetone in order to ensure dehydration, and then stored overnight, under vacuum, in equal parts of mixed acetone and polyester resin. They were then cleaned and set in a second batch of polyester resin. This reinforces the tooth to prevent shattering during the cutting phase of preparation. Three postcanines (UMZC T.480, UMZC T.485 and UMZC T.486; all *Diademodon* specimens) had previously been set in resin by an unknown method. These were sectioned using the same procedure as the other specimens, as described below.

The teeth in resin were cut through the marked plane using a low speed saw (Microslice II Annular Saw) with a 250 µm thick diamond wafering blade, to produce sections approximately 800 µm thick. To reduce their thickness, sections were mounted on microscope slides and lapped using a PM2A lapping machine (Logitech Materials Technologists Engineers) and 3 µm alumina powder until a thickness suitable for viewing under a polarized light microscope (approx. 100 µm depending on the section) was attained. A 1 µm diamond polish was used to finish lapping the sections.

The sections were bathed in soapy water to remove any remaining polish and cleaned in an ultrasonic bath of distilled water. Each section was zero-bonded to the surface of a clean glass microscope slide using a UV-curing resin. Following further cleaning in an ultrasonic bath, the sections were cleared using Histoclear^®^. Finally, the coverslip was mounted using Histomount^®^, a histological mounting medium.

### 2.3. Imaging and measurements

Sections were visualized and photographed by transmitted linear polarized light microscopy using an Olympus BX51 microscope mounted with a Q-Imaging Micro-Publisher 3.3 RTV camera, or a Zeiss Axioplan 1 microscope mounted with a Canon A630 camera. Measurements were taken using ImageJ 1.46 and Improvision Openlab 5.0.2 image analysis software.

**Figure 3.**
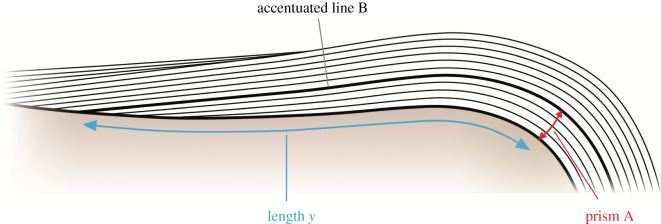
Quantifying CER. Black lines indicate long-period incremental lines of the enamel. The distance along a prism, A, from the EDJ to a particular accentuated line, B, was divided by the average cross-striation length (DSR) along that prism to yield *x* days to form that length of prism. By following the accentuated line B (which represents the forming front of enamel at a particular time) back to its intersection with the EDJ, the length of EDJ, *y*, from its intersection with stria B and its intersection with prism A can be measured. Length *y* therefore represents the increase in EDJ length after *x* days. Daily extension rate is, therefore, *y*/*x*. Selecting a new prism, intersecting the EDJ at the same point as stria B, allows the process to be repeated in an additive manner along the entire length of the EDJ.

The periodic enamel increments in the non-mammalian cynodont specimens and in crown mammal and diapsid specimens were used to calculate two important parameters of enamel growth rates: daily secretion rate (DSR), the distance measured between two daily lines, or the length of one daily increment, and crown extension rate (CER). CER is determined by the rate of ameloblast differentiation along the EDJ at the time of molar formation [12,13,72,103] and was calculated for crown mammals using the method of cumulative prism lengths described by Risnes [104] and modified by Dirks *et al*. [12], illustrated in figure 3. In our calculations in crown mammals, we have assumed that cross-striations are daily increments and striae of Retzius are long-period increments. The distance along a prism from as close as possible the tip of the dentine horn to a particular accentuated stria was measured. The mean DSR in this location was calculated by measuring cross-striation lengths along this prism and closely neighbouring prisms. Dividing the length of the prism from the EDJ to the accentuated line by the mean DSR yields *x* days to form that length of prism (figure 3). The accentuated line (which represents the forming front of enamel at a particular time) was followed back to its intersection with the EDJ, and the length of EDJ (*y*, μm) from this intersection to the tip of the dentine horn was measured. Length *y*, therefore, represents the increase in EDJ length after *x* days. The daily extension rate is, therefore, *y*/*x*. Starting with a new prism intersecting the EDJ at the same point as the first accentuated line, this entire process was repeated multiple times along the entire length of the EDJ. This allowed the CER and the DSR at different locations of the crown, from cusp to cervix, to be calculated. The average CER from cusp to cervix was calculated as the total length of EDJ measured (Σ*y*) divided by the total number of days (Σ*x*). The average DSR for this length of enamel was calculated as the overall mean cross-striation length measured. In those sections in which measurements were taken from more than one cusp, a grand average CER and a grand average DSR were taken for the entire tooth.

Increments of differing periods are clearly visible in the enamel of the non-mammalian cynodonts and crocodilians, but differ somewhat from the increments of extant mammals, more closely resembling the laminations found in the prismless enamel of extant mammals [3]. We identified lines equivalent with daily cross-striations and striae of Retzius (see the Results section for this interpretation), which in non-mammalian cynodonts we will refer to as ‘short-period incremental lines’ and ‘long-period incremental lines', respectively. We used these increments in an equivalent manner to the cross-striations and striae of Retzius in extant mammals to calculate the average DSR and the average CER for all teeth, from animals in all groups. Since it is difficult to follow the path of a single ameloblast in prismless enamel, such as in that of the non-mammalian cynodonts or interprismatic enamel layers of crown mammals, DSR measurements were taken between daily short-period lines at an orientation perpendicular to the outer enamel surface, as this would be the most likely path taken by an ameloblast-producing prismless enamel.

### 2.4. Statistical analysis

The average DSR and the average CER for all the non-mammalian cynodont, extant mammal and diapsid species studied were compared with similar growth rate measurements from additional crown mammal and diapsid species (electronic supplementary material, S1) taken from the literature (electronic supplementary material, S2).

Body mass should be considered when assessing differences in growth rates between species because there is a strong relationship between adult body mass and rates of body mass growth in all major vertebrate groups, including mammals [105]. Larger crown mammals also have larger teeth and jaws than smaller mammals. The area of the first molar has an isometric or negatively allometric relationship with body length, which, in turn, is highly correlated with body mass. Larger mammals have first molars that are either relatively small or directly proportional to their body length, but allometric relationships vary between clades [106]. We expect that these relationships mean that DSR and CER, which contribute to tooth size and the rate of growth, may also be correlated with body mass. To account for the potential effect of differences in body mass on enamel growth rates between species, we performed phylogenetic generalized least-squares (PGLS) regressions of the log DSRs and log CERs of all species against log body mass. This phylogenetic technique allows the growth variables to be adjusted for body mass while also taking into account the non-independence of the data owing to the shared phylogenetic history of species [107,108]. The residuals from these PGLS regressions were then used as body mass-corrected DSR and body mass-corrected CER in further analyses. Body mass data for all species and details of the construction of the phylogenetic tree used in PGLS analysis can be found in electronic supplementary material, S3 and S4.

To compare differences in enamel growth rates between different non-mammalian cynodonts, crown mammals and diapsids, species were sorted into five different groups: mammaliamorphs (*Morganucodon* and *Oligokyphus*); non-probainognathian cynodonts (*Thrinaxodon*, *Diademodon*, *Scalenodon* and *Massetognathus*); diapsids (*Crocodylus*, *Borealosuchus* and *Sphenodon*), hypsodont crown mammals (*Ovis aries* [Catalonian breed], *Ovis aries* [Soay breed] and *Myotragus*) and non-hypsodont crown mammals (all remaining crown mammal species). Hypsodont and non-hypsodont mammals were included as separate groups owing to the unusually high enamel growth rates in the former relative to the latter (electronic supplementary material, S1). It should be noted that while all non-mammalian cynodont species are also non-hypsodont, throughout this study the term ‘non-hypsodont mammals’ refer only to those taxa in the crown group. Figure 1 illustrates the phylogenetic relationships between diapsids, non-probainognathians, mammaliamorphs and crown mammals.

*Exaeretodon* was excluded from the analysis of these data because it showed a pattern of enamel deposition unlike any other group of amniotes, extinct or extant (see Results). *Irajatherium* appears to have a pattern of enamel growth intermediate between the non-probainognathians and mammaliamorphs. Hence, we conducted separate tests, including and excluding it in the mammaliamorph group or in the non-probainognathian group, in order to probe its position.

Body mass-corrected DSR and body mass-corrected CER in the different groups were compared in a multivariate analysis of variance (MANOVA), following Box-Cox transformation of the variables for multivariate normality [109]. The MANOVA was performed using the statistical program R v. 3.4.4. MANOVA is a generalized form of ANOVA in which multiple dependent variables (transformed, body mass-corrected DSR and transformed, body mass-corrected CER) can be compared between different groups [110]. We undertook pairwise comparison of the different groups and adjusted *p*-values for multiple comparisons using false discovery rate (FDR) correction [111,112]. In addition, we conducted separate univariate ANOVAs on each of these variables [110]. For each variable, we performed pairwise comparisons of each group and corrected the significances of these using FDR.

The main analysis was performed using the average DSR and CER of all teeth sectioned from a species as a data point. Given the difficulty of obtaining rare fossil specimens for sectioning, relatively low numbers of taxa were included in the mammaliamorph and non-probainognathian groups. Owing to the small sample sizes of these groups, the Kruskal–Wallis test (a non-parametric analogue of ANOVA) was also carried out separately on DSR and CER values in order to assess differences between groups. Dunn tests [113] were used to perform pairwise comparisons, and *p*-values were adjusted for multiple comparisons using the FDR method.

## 3. Results

### 3.1. Enamel histology in non-mammalian cynodonts

**Figure 4.**
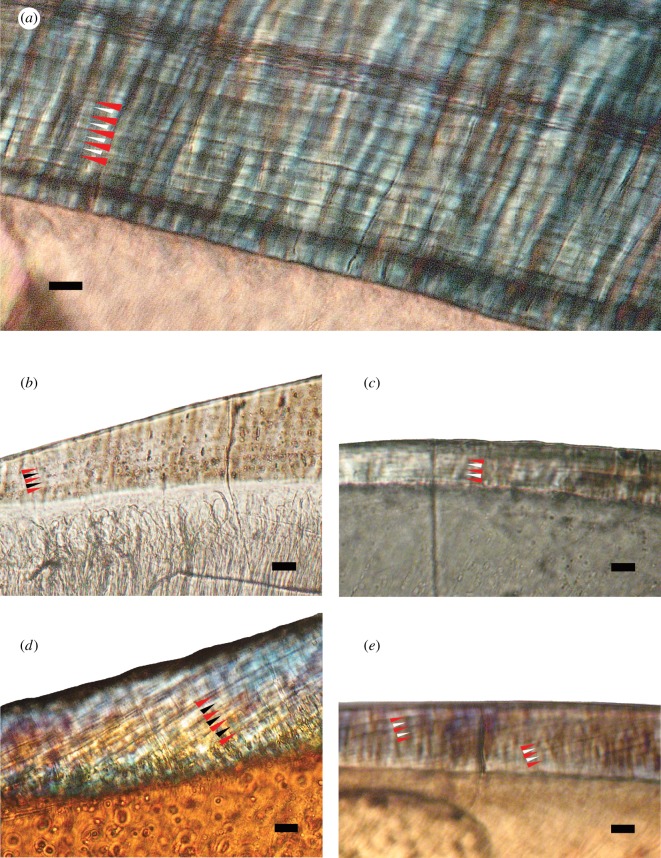
Polarized light microscope images of incremental lines in the enamel of postcanines of non-mammalian cynodonts. Red arrows indicate long-period incremental lines, and white (*a*,*c*,*e*) or black arrows (*b*,*d*) indicate short-period incremental lines. In all species of non-mammalian cynodont, there appears to be one short-period line between any two adjacent long-period lines.(*a*) *Diademodon* sp. (UMZC T.486), (*b*) *Massetognathus pascuali* (CRILAR-C 162.2), (*c*) *Irajatherium hernandezi* (UFRGS-PV-1348-T-S1), (*d*) *Oligokyphus* sp. (UMZC 1236a), (*e*) *Morganucodon watsoni* (UMZC 2017.4.1). Scale bars = 10 µm. Outer enamel surface to the top. Cervix to the left. Image (*e*) slightly out of focus to visualize both long- and short-period increments in the same image as they are not in precisely the same plane within the section.

We observed incremental lines in the enamel of all the non-mammalian cynodont species we investigated. The increments formed regularly spaced dark bands roughly parallel to the enamel surface and EDJ, although in some species, notably the mammaliamorphs *Morganucodon* and *Oligokyphus*, these bands formed a somewhat steeper angle with the EDJ. These prominent dark bands are henceforth referred to as ‘long-period incremental lines’ and are marked with red arrows in figure 4. Fainter, dark sub-increments are visible approximately at half the distance between pairs of long-period lines. These are referred to as ‘short-period incremental lines’ and are marked with black or white arrows in figure 4. In all the cynodont species observed, one long-period increment (the distance between two successive long-period lines) is composed of two short-period increments (the distance between a short-period line and a long-period line).

**Figure 5.**
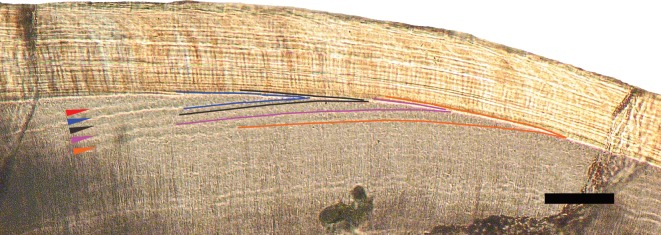
Polarized light microscope image of incremental lines in the enamel (top) and dentine (bottom) of a postcanine of the non-mammalian cynodont *Diademodon* sp. Coloured arrows indicate daily lines of von Ebner in the dentine. Incremental lines in both the enamel and the dentine can be seen along the same length of EDJ in this specimen, and lines of the same colour indicate equivalent daily lines in the dentine and enamel. Scale bar, 100 µm. Cervix to the right.

The incremental lines in cynodont species (figure 4) differ from those most commonly observed in extant mammals under light microscopy (e.g. fig. 4 in [3]). Columnar cynodont enamel lacks prisms, resulting in the absence of prism cross-striations, which usually cross prisms perpendicularly in extant mammals [3]. However, cynodont incremental lines do strongly resemble the laminations which may be found in the prismless surface enamel of many extant mammals (fig. 8 in [3]). These laminations have similar spacing to cross-striations [3,15,82]. However, unlike cross-striations, laminations follow a course roughly parallel to the striae of Retzius [14,15,80–82,91], which form successive dome-like layers over the dentine horn in the cuspal enamel and extend towards the cervix, terminating at the outer enamel surface in lateral enamel [5,78,93]. The incremental lines in cynodont enamel also follow such a course (figures 4 and 5). We suggest that the prominent, dark long-period lines observed in cynodont enamel correspond with the striae of Retzius of extant mammals, while the fainter short-period lines between them are homologous with laminations, in other words, that there is a 2-day periodicity between the long-period lines, with short-period lines representing 1 day of enamel secretion.

In the Discussion, we consider the possibility that the dark long-period lines actually have a 1-day periodicity (with the short-period lines being sub-daily), but conclude that this is unlikely. Thus, unless stated otherwise, in all statistical analyses discussed below, we base our calculations of DSR and CER in cynodont enamel on the assumption that long-period incremental lines have a 2-day periodicity.

### 3.2. Differences in enamel growth rates between groups

**Figure 6.**
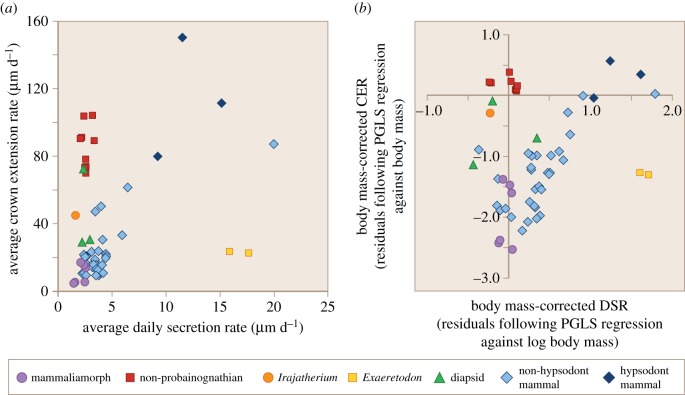
Enamel growth rates from different individual teeth belonging to different amniote groups, assuming a 2-day periodicity between long-period lines in non-mammalian cynodonts. (*a*) Raw growth rates: average DSR (μm d^−1^) versus average CER (μm d^−1^). (*b*) Body mass-corrected growth rates: the residuals of log DSR (µm d^−1^) and log CER (µm d^−1^) following phylogenetic generalized least-squares regression against log body mass. The growth rates of individual teeth were included as separate data points, rather than using the species average.

Figure 6 shows the differences between enamel deposition rates in different groups. The majority of non-hypsodont crown mammals have a low DSR and CER, producing relatively thin layers of enamel each day, which accrete gradually to form the crown of the tooth. By contrast, hypsodont mammals develop their high-crowned teeth both by secreting thicker layers of enamel each day and extending enamel more rapidly along the EDJ. Diapsids have low DSRs similar to those of non-hypsodont mammals, but have somewhat higher CERs. Among cynodonts, the mammaliamorphs do not differ greatly from the non-hypsodont mammalian pattern (although they do have relatively low DSRs in comparison to non-hypsodont mammals), while non-probainognathian cynodonts differ in their much higher CERs. Indeed, the CER values of most non-probainognathians exceed those of diapsids and are comparable to those of hypsodont crown mammals. However, non-probainognathians lack hypsodonty themselves and differ from high-crowned mammals in having low DSR values, more similar to those of mammaliamorphs and non-hypsodont mammals. Thus, non-probainognathians resemble hypsodont mammals in having a high CER, but resemble non-hypsodont mammals in having a low DSR. *Irajatherium*, a probainognathian tritheledontid, shares a low DSR with all other cynodont species, but has a CER intermediate between the rates of non-probainognathians and of mammaliamorphs. The derived traversodontid *Exaeretodon* differs from all groups, extinct and extant in having both a low CER and a high DSR.

Following correction for body mass in a phylogenetic context and Box-Cox transformation, a MANOVA of DSR and CER across the five groups of amniote investigated (non-hypsodont crown mammals, hypsodont crown mammal, diapsid, mammaliamorph and non-probainognathian cynodont) is highly significant (Wilks' statistic = 0.169, *F*_8, 66_ = 11.8, *p* < 0.001) and confirms these differences between groups. Pairwise group comparison shows that most groups differ significantly from one another (table 2), although diapsids show some overlap with other groups. Notably, both mammaliamorphs (*p* = 0.0163) and non-hypsodont mammals (*p* < 0.001) differ from non-probainognathians (figure 6). Univariate ANOVAs performed separately on body mass-corrected and transformed DSR and CER were highly significant (table 3), and pairwise comparisons show that the difference between non-probainognathians and mammaliamorphs and between non-probainognathians and non-hypsodont mammals is accounted for by differences in CER. Non-probainognathians show a significant difference from mammaliamorphs with respect to CER (*p* = 0.0051; table 3), but not DSR (*p* = 0.5200; table 3), and a significant difference from non-hypsodont mammals with respect to both growth measures (table 3). Mammaliamorphs differ from non-hypsodont mammals with respect to DSR (*p* = 0.0189; table 3), but their CERs do not differ significantly (*p* = 0.4438; table 3). Hypsodont mammals have significantly greater DSRs than all other groups. In summary, non-probainognathians have a greater CER than either mammaliamorphs or non-hypsodont mammals, while mammaliamorphs have a low CER, similar to that of non-hypsodont mammals. Both cynodont groups (mammaliamorphs and non-probainognathians) have a lower DSR than non-hypsodont mammals.

**Table 2.** Analysis of the differences between five groups of amniotes in two enamel growth rate variables, DSR and CER. DSR and CER were calculated assuming a 2-day periodicity between long-period lines in non-mammalian cynodonts. MANOVA comparing DSR and CER (both body mass-corrected using phylogenetic generalized least-squares regression and transformed) across the five groups, with pairwise comparison *p*-values adjusted by FDR. *Exaeretodon* and *Irajatherium* were excluded from this analysis.

effect	Wilks' *λ*	*F*-value	degrees of freedom	*p*-value	partial *η*^2^ (effect size)
group	0.169	11.834	8,66	0.000000	0.589

	diapsid	non-probainognathian	mammaliamorph	non-hypsodont mammal	
non-probainognathian	0.1574	—	—	—	
mammaliamorph	0.1944	0.0163^a^	—	—	
non-hypsodont mammal	0.0006^a^	0.0000^a^	0.0448^a^	—	
hypsodont mammal	0.0732	0.0059^a^	0.0713	0.0059^a^	

^a^Significant differences between groups at *α* < 0.05.

**Table 3.** Analysis of the differences between five groups of amniotes in two enamel growth rate variables, DSR and CER. DSR and CER were calculated assuming a 2-day periodicity between long-period lines in non-mammalian cynodonts. Separate univariate ANOVAs on DSR and CER (both body mass-corrected using phylogenetic generalized least-squares regression and transformed) across the five groups, with pairwise comparison *p*-values adjusted by FDR. *Exaeretodon* and *Irajatherium* were excluded from this analysis.

dependent variable	sum of squares	mean squares	degrees of freedom	*F*-value	*p*-value (FDR-adjusted)	partial *η*^2^ (effect size)
DSR	0.209	0.052	4,34	10.75	0.00001938	0.558
CER	0.005	0.001	4,34	7.578	0.00017700	0.471

	diapsid	non-probainognathian	mammaliamorph	non-hypsodont mammal		
*DSR (body mass-corrected and transformed)*						
non-probainognathian	0.7526	—	—	—		
mammaliamorph	0.8900	0.5200	—	—		
non-hypsodont mammal	0.0168^a^	0.0162^a^	0.0189^a^	—		
hypsodont mammal	0.0189^a^	0.0042^a^	0.0162^a^	0.0046^a^		
*CER (body mass-corrected and transformed)*						
non-probainognathian	0.0800	—	—	—		
mammaliamorph	0.1255	0.0051^a^	—	—		
non-hypsodont mammal	0.1471	0.0046^a^	0.4438	—		
hypsodont mammal	0.1667	0.7078	0.0168^a^	0.0122^a^		

^a^Significant differences between groups at *α* < 0.05.

Results from non-parametric Kruskal–Wallis tests (electronic supplementary material, S5.5), are consistent with those obtained using MANOVA: non-probainognathians differ from both mammaliamorphs (*p* = 0.0153) and from non-hypsodont mammals (*p* = 0.0060) with respect to CER, but non-hypsodont mammals do not differ from mammaliamorphs in CER (*p* = 0.2616).

All results reported above assume a 2-day periodicity between long-period lines (short-period striations are assumed to be daily), as we consider this more likely than a possible alternative interpretation of a 1-day periodicity between long-period lines (long-period lines form in 1 day, with the short-period striations being sub-daily). However, in order to test the effect of this assumption, we also calculated the DSR and the CER for all non-mammalian cynodont species assuming a 1-day periodicity of long-period lines and repeated statistical tests under this assumption. As expected, under a 1-day periodicity assumption, daily increments are twice as long as under a 2-day periodicity assumption, and thus, the CER is also roughly doubled, because the time taken to cover a particular length of the EDJ is halved.

Assuming a 1-day periodicity yields a MANOVA which is also highly significant (Wilks' statistic = 0.205, *F*_8, 66_ = 9.99, *p* < 0.001), and the significance of pairwise comparisons between non-probainognathians, mammaliamorphs and non-hypsodont mammals is the same as for the 2-day periodicity assumption with respect to the important comparisons between non-probainognathians, mammaliamorphs and non-hypsodont mammals (electronic supplementary material, S6). Non-probainognathians differ from both mammaliamorphs (*p* = 0.0021) and from non-hypsodont mammals (*p* = 0.0001) with respect to CER; non-hypsodont mammals do not differ from mammaliamorphs with respect to CER (*p* = 0.3847). The only potentially important difference under the 1-day periodicity assumption is that non-hypsodont mammals do not differ from mammaliamorphs in DSR (*p* = 0.5489), while there is a difference between the two groups with respect to DSR under a 2-day periodicity assumption.

*Exaeretodon* has a very unusual pattern of enamel growth, with a high DSR, but a low CER, and appears to differ from all the groups included in this study (figure 6*a*). It was therefore excluded from the statistical comparisons presented here.

*Irajatherium*, a probainognathian tritheledontid, appears to occupy an intermediate position between the non-probainognathian group and the other probainognathians, *Morganucodon* and *Oligokyphus*. It has a CER of 44.7 µm d^−1^, compared to a mean CER of 86.0 µm d^−1^ in non-probainognathians and 10.1 µm d^−1^ mean CER in mammaliamorphs. Repeating the MANOVA including *Irajatherium* with mammaliamorphs in a ‘probainognathian group’ results in this probainognathian group still showing a significant difference from non-probainognathians with respect to CER and no significant difference from non-hypsodont mammals with respect to CER (electronic supplementary material, S7.1.2). Alternatively, including *Irajatherium* in the non-probainognathian group also results in a significant difference between non-probainognathians and mammaliamorphs with respect to CER and no significant difference from non-hypsodont mammals with respect to CER (electronic supplementary material, S7.2.2). These similar results, regardless of the group in which *Irajatherium* is placed, reflect the intermediate enamel growth patterns of *Irajatherium* relative to non-probainognathians and mammaliamorphs.

## 4. Discussion

We observed periodic incremental lines in histological thin sections of the enamel of all the cynodont species in this study. This confirms previous observations of the presence of incremental bands in the enamel of *T. liorhinus* [27], *Diademodon* sp. [27], *Massetognathus* sp. [28] and *E. riograndensis* [29], and provides evidence for similar increments in the other species investigated (*S. angustifrons*, *I. hernandezi*, *Oligokyphus* sp. and *M. watsoni*). We observed both short- and long-period increments in cynodont enamel (figure 4) and consider these to be equivalent to (respectively) the daily laminations and striae of Retzius of extant mammalian enamel. The calculation of enamel growth rates from these increments shows that there is a very pronounced difference in CER between more stemward non-probainognathian cynodonts and more crownward mammaliamorphs.

### 4.1. The nature and periodicity of laminations in non-mammalian cynodont enamel

The appearance of daily markings as laminations rather than cross-striations in cynodont enamel is consistent with current hypotheses concerning both the formation of laminations and the microstructure of non-mammalian cynodont enamel (figure 2). Smith [3] suggests that the entire enamel-forming front of secretory ameloblasts is involved in the circadian rhythm producing laminations, which therefore have a different topographic origin from cross-striations. This is supported by evidence that laminations, although not restricted to prismless enamel in extant mammals, are frequently found to be prominent in prismless regions close to the EDJ and the outer enamel surface [3,84,99]. These locations are formed either before development of the distal portion of the Tomes' process or after its regression. Hence, cross-striations, which are produced perpendicular to prisms by one face of the distal Tomes' process, are not formed [15,72].

Daily markings in these prismless regions instead take the form of laminations (figure 2, left panel), produced from more proximal interprismatic enamel growth regions of the ameloblast [15]. With the possible exception of *Pachygenelus* [50], the enamel of all the non-mammalian cynodonts investigated to date is prismless [20,76,77]. Their ameloblasts lacked Tomes' processes and enamel was produced across a flat secretory surface [71], which would preclude the formation of cross-striations from distal secretory regions of the ameloblast. Thus, the fact that daily markings take the form of laminations in non-mammalian cynodonts is expected, given the hypothesized processes of enamel development in these animals.

Uncertainty about 1- versus 2-day periodicity in enamel lamination is worth comment. If the darker, long-period lines (figure 4) have 1-day periodicity, then the fainter, short-period lines are sub-daily; if they represent a 2-day periodicity, then short-period lines are daily. Striae of Retzius with multiple daily laminations between them have been observed: for example, macaques have a 5-day periodicity between Retzius lines [84], while fossil and extant rhinocerotids have a 7-day periodicity [81]. By contrast, enamel in which supra-daily lines are not observed (i.e. striae of Retzius are indistinguishable from laminations with a 1-day periodicity) has also been shown to occur in other extant mammal groups such as cervines [80], caprines [14] and suids [15].

In the enamel of domesticated dwarf *Sus scrofa*, which have periodic laminations of 1 day, darker and broader incremental bands are successively followed by thinner and lighter continuous incremental lines (Fig. 5 in [15]). Kierdorf *et al*. [15] suggest that these features are formed simultaneously, with the darker, broader bands representing the forming front of prismatic enamel produced by the distal Tomes' process and the lighter incremental lines representing the forming front of interprismatic enamel produced by the proximal part of the Tomes' process. It could be possible to misinterpret these simultaneously produced, yet spatially offset daily features; if the darker bands were designated as striae of Retzius and the lighter as daily markings, a 2-day periodicity between the darker bands could be erroneously surmised.

We consider it unlikely that such a situation has occurred in the non-mammalian cynodont enamel. The long-period incremental lines and short-period incremental lines do not form successive dark and light bands, but rather both types of lines are distinct from, and darker than, the surrounding matrix against which they appear (figure 4). Furthermore, the ameloblasts producing the prismless synapsid columnar enamel of these cynodont species lacked Tomes' processes, and probably had flat secretory surfaces [71], so this mechanism of producing simultaneously formed, spatially offset increments is extremely unlikely in non-mammalian cynodonts.

Nevertheless, markings formed by sub-daily oscillation in ameloblast activity are also occasionally visible between laminations [3,14,15], and misidentification of such sub-daily markings for daily laminations could lead to underestimation of enamel growth rates. However, these sub-daily markings are described as strongly resembling cross-striations [14,15], and this is not the case for the short-period incremental lines of cynodont enamel, which are laminar in appearance.

As a test of 1-day versus 2-day periodicity of the long-period incremental lines, we compared the extension rates of enamel along the EDJ, assuming different periodicities, with extension rates of dentine along the same length of EDJ in *Diademodon* (figure 5). The dentine of *Diademodon* (and indeed all the other non-mammalian cynodonts in which increments were observed) shows increments (figure 5) strongly resembling the lines of von Ebner which are found in a broad taxonomic range of extant vertebrates [17,114,115], extinct dinosaurs [18,26] and extinct synapsids [16,23–25].

Periodic labelling in extant taxa shows that these lines accumulate daily [17,115]. They appear as alternating light and dark bands under polarized light [18] with an average width of 16 µm in small mammal species [115], although smaller widths of 4 µm are known for humans and macaques [115]. Of previously studied non-mammalian synapsids, lines of von Ebner are approximately 20 µm in *Diictodon* [16], 13.7 µm in *Tritylodon* [23], 17.4 µm in *Lystrosaurus* [24] and 18.8 µm in an unnamed dicynodont [25]. The average width of the increments we measured in *Diademodon* (11.9 µm) is somewhat narrower than in these other synapsids. However, data from the three dicynodont species are from enlarged tusks which may be expected to show greater rates of growth, and *Diademodon* dentine increment widths fall well within the range of von Ebner line widths of other extinct and extant amniote taxa. We therefore consider them to represent daily dentine deposition in common with other amniotes.

We calculated the average extension rate along the EDJ of *Diademodon* specimen UMZC T.485 using dentine lines of von Ebner in an equivalent manner to the method described for using daily enamel increments, and found it to be 88.3 µm d^−1^. The average extension rate along the same length of EDJ, but using enamel increments instead of dentine, yielded 179.2 µm d^−1^ assuming a 1-day periodicity of long-period incremental lines and 89.3 µm d^−1^ assuming a 2-day periodicity. Clearly, the enamel and dentine extension rates are closely in accordance under the 2-day periodicity of enamel assumption, but not under the 1-day periodicity assumption.

Therefore, because the short-period lines we observe in non-mammalian cynodont enamel do not greatly resemble the sub-daily markings between laminations, and because assuming a 2-day periodicity of long-period lines yields extension rates along the EDJ more in accord with those found using dentine daily increments, we conclude that the short-period and long-period incremental lines of cynodont enamel are, respectively, the equivalents of daily laminations and striae of Retzius in extant mammals, and that long-period incremental lines have 2-day periodicity across all non-mammalian cynodonts studied.

Why the periodicity of long-period increments should be 2 days consistently across the non-mammalian cynodonts is unclear; it would certainly be interesting to investigate a greater taxonomic sample of cynodonts and more basal synapsids to establish how widespread this characteristic may be. In addition, future studies could further test this result by focusing on periodic vital labelling of, for example, crocodilian or lepidosaur teeth in order to improve our understanding of periodicity in extinct and extant non-mammalian amniotes.

### 4.2. Differences in enamel growth rates of non-mammalian cynodonts

There is a striking difference in enamel growth rates between more stemward non-probainognathian cynodonts and the more crownward mammaliamorphs, *Oligokyphus* and *Morganucodon*. This is demonstrated in a MANOVA of DSRs and CERs, corrected by body mass in a phylogenetic context, in which mammaliamorphs are found to differ significantly from non-probainognathians, but not from non-hypsodont crown mammals in their CERs, suggesting that a more placental mammal-like pattern of enamel extension was present in mammaliamorphs. DSR is lower in both non-probainognathian and mammaliamorph groups in comparison to non-hypsodont mammals, although the difference is much less marked than the difference in CER between non-probainognathians and mammaliamorphs; the average DSR of the non-hypsodont mammals is approximately twice that of the cynodont groups, while CER is approximately 10 times as fast in non-probainognathians in comparison to mammaliamorphs (electronic supplementary material, S5.2). Assuming a 1-day periodicity of the long-period increments yields the same result regarding differences in CER between non-hypsodont mammals, non-probainognathians and mammaliamorphs (electronic supplementary material, figure S6), although the differences in DSR between the cynodont groups and non-hypsodont mammals are not significant under this assumption.

**Figure 7.**
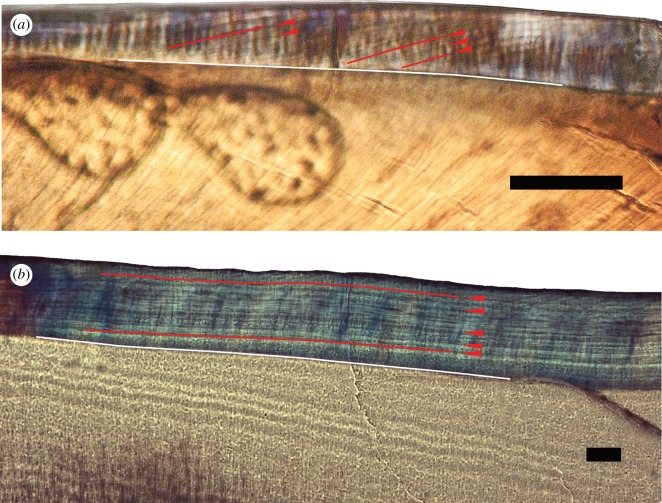
Polarized light microscope images of incremental lines in the enamel of postcanines of the mammaliamorph *M. watsoni* UMZC 2017.4.1 (*a*) and the non-probainognathian *Diademodon* sp. UMZC T.486 (*b*). Red arrows indicate prominent long-period incremental lines, with the paths of some traced for emphasis. White lines indicate the EDJ. Long-period lines of *Morganucodon* approach the EDJ at a steeper angle of inclination, indicating more gradual accretion of enamel than in *Diademodon*, in which enamel is rapidly extended, causing long-period lines to approach the EDJ at a very small angle. Scale bars, 50 µm. Cervix to the left in both images.

In Figure 6*a*,*b*, separate univariate ANOVAs (table 3) and Kruskal–Wallis tests (electronic supplementary material, S5.5) on DSR and CER show the major difference between non-probainognathians and mammaliamorphs to be in the much greater CERs of non-probainognathians. The relatively slow CERs of mammaliamorphs are reflected in the large angle by which long-period lines approach the EDJ (figure 7*a*). A larger angle of inclination indicates a more gradual accretion of enamel from cusp to cervix, more similar to that seen in extant mammals; a shorter length of EDJ has been covered by enamel over a particular period of time ([103]; figure 3). This contrasts with the appearance of non-probainognathian enamel, in which long-period lines form a very small angle with the EDJ (figure 7*b*), indicating that enamel has been laid in very rapidly extending sheath-like layers across large areas of the crown surface, rather than by gradual accretion. The difference in CER between these groups is very pronounced, with a mean CER in non-probainognathians over eight times more rapid than mammaliamorphs. Indeed, the CER values of non-probainognathians are more comparable with those of the extremely rapidly extending hypsodonts than with either mammaliamorphs or non-hypsodonts (table 3 and figure 6).

### 4.3. Enamel extension rates and skeletal growth in non-mammalian cynodonts

We suggest that this very pronounced difference in CERs between mammaliamorphs and non-probainognathians relates to the changes in skeletal growth pattern and dental replacement that occurred during the origin of mammaliaforms (figure 1).

One important aspect of this transition is the origin of a reduced diphyodont pattern of dental replacement, which is observed most basally in the mammaliaform *Morganucodon* [33,36,37,42]. Both rapid juvenile growth and determinate truncated adult growth restrict the time at which the jaws are at an intermediate size, and thus, the evolution of these growth characteristics are likely a necessary condition for the evolution of reduced diphyodont tooth replacement [32,36,46–48]. Mammal-like skeletal growth patterns are indeed observed in more crownward non-mammalian cynodonts such as *Morganucodon* and, to some extent, in *Oligokyphus* [36,46,62], while it is likely that more stemward non-mammalian cynodonts had more extended, less determinate adult growth periods [36,37,116]. Bone microstructure studies show that growth is not ‘indeterminate’ in these more stemward cynodont species (because growth does reduce in rate and eventually ceases in adulthood), and that there is variability in growth strategies of non-mammalian cynodonts and therapsids [57,58,117]. However, overall, there is a trend towards more rapid, mammal-like bone deposition in more crownward groups [57,58,118].

The more mammal-like growth patterns of the mammaliamorphs *Morganucodon* and *Oligokyphus*, in comparison with more stemward non-probainognathians, may account for the differences observed in CER of the enamel of these groups. There is a connection between rates of enamel growth and skeletal bone growth; Bromage *et al*. [9] demonstrated that a single bone lamella forms during the same time period as the repeat interval (number of days of formation) of the enamel striae of Retzius. They suggest that this time interval reflects a fundamental rhythm, termed the Havers–Halberg oscillation, which regulates osteoblast proliferation and secretion, setting the pace of skeletal growth. This rhythm then has a secondary effect on ameloblast activity and enamel formation rates, resulting in coupling between enamel and bone growth [9].

While Bromage *et al*. [9] did not specifically investigate the relationship between bone growth and enamel extension rate (which relates to ameloblast differentiation and proliferation along the EDJ), similar coupling between bone osteoblast and ameloblast differentiation and proliferation may be plausible. This possibility of a link between skeletal growth and enamel extension rates is supported by the much higher extension rates of deciduous teeth in comparison with permanent teeth in extant mammals [103,119], which could be explained as a reflection of higher underlying rates of bone growth at the time of deciduous tooth formation. Shellis [103] shows that, in humans, the enamel extension rate of deciduous teeth (averaged across all different tooth types) is approximately five times higher than the extension rate of permanent teeth (averaged across all different tooth types). The effect is even more pronounced when deciduous extension rates are compared with only the permanent third molar; in this case, average deciduous enamel extension rates are approximately eight times greater [103]. The permanent third molar forms during late adolescence or early adulthood when underlying somatic growth rates are considerably slowed or have ceased, perhaps resulting in concomitantly greatly reduced CERs.

A similar effect may account for the difference in enamel extension rates observed between non-probainognathians and mammaliamorphs. The lower CERs of mammaliamorphs may suggest that underlying skeletal growth rates were much reduced at the time of postcanine formation due to more determinate patterns of adult growth, while non-probainognathians may not have ceased skeletal growth so abruptly, resulting in higher underlying growth rates during postcanine formation, even in large individuals. While this may be a plausible explanation of the differences between these two groups of non-mammalian cynodonts, further information on the relationship between enamel CERs and bone growth in extant species would be required to support this hypothesis. Additionally, it could be interesting to investigate enamel growth rates in deciduous teeth of *Morganucodon*, or in earlier generations of teeth in more stemward non-mammalian cynodonts; these could potentially permit construction of a profile of dental growth rates at different stages of development within a species, which could lead to greater understanding of the origin of determinate skeletal growth patterns.

### 4.4. Enamel extension rates and dental replacement in non-mammalian cynodonts

Although we argue that differences in underlying skeletal growth pattern may give rise to differences in enamel extension rate, we do not think that it fully accounts for the very large differences between groups. Non-probainognathians have extension rates on average 10 times greater than in mammaliamorphs, and yet the difference in adult skeletal growth patterns was unlikely to have been quite so pronounced between these groups. Bone histological studies of many therapsids suggest that non-probainognathian skeletal growth was generally asymptotic and ceased in adult animals [55,58,59,120], even if not necessarily so abruptly as in *Morganucodon*. Thus, adult non-probainognathians would also be expected to show reduced growth rates, and the enamel extension rates of non-probainognathians may not be expected to differ from those of adult mammaliamorphs to the extreme observed in this study. Therefore, while we certainly do not exclude underlying skeletal growth as a, possibly important, contributing influence on enamel growth, it seems unlikely that underlying growth differences are the sole explanation for differences in enamel extension between non-probainognathians and mammaliamorphs. Changes in tooth replacement rates relating to the origin of diphyodonty (figure 1) may also explain the differences observed in this study between the two groups of cynodonts. Reduction in the number of dental replacements in mammaliamorphs (in comparison with the majority of non-probainognathians) may have permitted a longer growth period of the tooth row, and hence, slower rates of crown extension in individual teeth.

Diphyodont tooth replacement, in which antemolars are replaced only once and molars are unreplaced, occurs in nearly all extant mammals and most basally in *Morganucodon* [33,36,37,42]. Most non-mammalian cynodonts stemward of *Morganucodon*, including, for example, *Thrinaxodon* [61] and *Pachygenelus* [121], have polyphyodont, alternate replacement from anterior to posterior along the tooth row, often with non-replacement of the anterior postcanines [36,37,61,116,122]. Gomphodontian cynodonts show a variety of dental crown morphologies and replacement modes. An interesting specialized form is *Diademodon* in which the number of replacements is reduced, although not to the same extent as in diphyodont mammals [123]. The anterior postcanines are not replaced, but are shed to form a diastema, with new teeth erupting at the distal end of the tooth row to maintain tooth number. Other teeth are replaced by those of more mesial morphological characteristics [60,123]. However, this highly derived and unusual condition probably relates to maintaining occlusion of their differentiated gomphodont dentition during growth, rather than to the origin of mammalian growth patterns [33,60,123,124]. Regardless, posterior postcanines are still replaced in *Diademodon* [123], as they are in basalmost traversodontid taxa, such as *Andescynodon* [125] and *Boreogomphodon* [126]. However, a reduction in dental replacements is observed in more derived traversodontids; notably, taxa more derived than *Massetognathus*, such as *Exaeretodon*, show no replacement of the postcanines, but new postcanines are added to the toothrow distally and lost mesially [127,128]. *Exaeretodon* also loses postcanines during its development towards adulthood [128]. Replacement mode is therefore variable among non-probainognathians. While derived traversodontids, such as *Exaeretodon*, show no vertical replacement of the postcanines, the majority of members of the group used here (i.e. non-probainognathian cynodonts) have multiple replacements of postcanines, with this trait being most pronounced in the basal forms, such as *Thrinaxodon* [61].

In more crownward taxa such as tritylodontids (e.g. *Oligokyphus*), replacement is reduced, in a similar manner to the condition of *Exaeretodon*, such that postcanine teeth are not replaced at all, but discarded mesially and added distally with a mesial displacement of the tooth row [36,48,129]. This reduction in dental replacement (albeit not to diphyodonty) in tritylodontids may be permitted by their somewhat more mammal-like skeletal growth patterns; growth in *Oligokyphus* differed from the patterns of non-avian diapsids and approached the condition of *Morganucodon* or extant placental mammals, although it probably had a more extended period of adult growth [46].

The variability in modes of replacement among non-probainognathians, particularly between *Thrinaxodon* [61], and ‘partially’ sectorial toothed cynodonts with gomphodont dentitions such as *Diademodon* [60,123], and also *Andescynodon* [125] and *Boreogomphodon* [126], make direct comparison of the numbers of replacements in each species difficult. However, it is clear that the greatest numbers of replacements occur in the basal epicynodont *Thrinaxodon*, with relatively high replacement also observed in *Diademodon*. The greatest reductions in replacement are seen in the two mammaliamorph species, *Morganucodon* and *Oligokyphus* [36,48,129], and in the derived traversodontid *Exaeretodon* [127,128]. As *Exaeretodon* was excluded from the main analysis, the mammaliamorph group represents a reduction in dental replacement in comparison with most members of the non-probainognathian group in this analysis.

The relatively higher rates of replacement in more stemward non-probainognathians may explain the need for higher rates of crown extension (e.g. *Thrinaxodon*: mean CER = 91.1 µm d^−1^) in these animals; higher rates of crown extension would reduce overall tooth development time, allowing relatively more frequent replacements at a particular locus. Among more crownward mammaliamorphs, replacement rates are reduced, permitting longer overall development times for the dentition, and a decrease in CERs (*Morganucodon*: mean CER = 4.8 µm d^−1^; *Oligokyphus*: mean CER = 12.8 µm d^−1^). Notably, among non-probainognathians, *Exaeretodon*, which also has reduced postcanine replacement, crown extension is also low (mean CER = 22.7 µm d^−1^), while *Thrinaxodon* (mean CER = 91.1 µm d^−1^) and *Diademodon* (mean CER = 98.8 µm d^−1^) have the greatest rates of crown extension (*Thrinaxodon* has the greatest extension rate when adjusted for body size). This supports a link between dental replacement and CERs.

#### 4.4.1. Irajatherium

The tritheledontid *Irajatherium* appears to have rates of enamel extension somewhat intermediate between those of non-probainognathians and mammaliamorphs (figure 6). This suggests that this species may have had an intermediate pattern of skeletal growth, or reduction in dental replacement relative to non-probainognathians such as *Thrinaxodon* [61], or both.

Preliminary studies on bone histology in *Irajatherium* show a rapid early growth with annual interruptions later in ontogeny [118], which may be consistent with an intermediate pattern of skeletal growth. Tooth replacement data in *Irajatherium* are still limited, but it has been shown to have the alternate postcanine replacement with loss of the anterior postcanines typical of many more stemward non-mammalian cynodonts [130,131]. To assess whether the numbers of replacements are reduced relative to more stemward non-mammalian cynodonts would require investigation of additional material. Thus, a further study of dental replacement rates and growth in *Irajatherium* could serve as a useful test of our hypothesis regarding the link between enamel extension rates, skeletal growth and dental replacement in non-mammalian cynodonts.

In figure 1, we follow the recent dated phylogeny of Ruta *et al*. [31], in which Tritylodontidae is resolved as the sister group to Mammaliaformes, with brasilodontids and ictidosaurians forming successive outgroups (but see also [132,133]). In this phylogeny, the intermediate crown development of the tritheledontid *Irajatherium* appears to be part of a trend from rapidly extending enamel in more stemward taxa towards the gradually accreting enamel of mammaliamorphs. If, however, ictidosaurians are resolved in a closer relationship to mammaliaforms [134–136], then there may be no general trend towards gradual reduction in enamel extension rates in successively more crownward groups, but possibly separate reductions in tritylodontids and mammaliaforms. Nevertheless, the entire probainognathian group would represent a reduction in enamel extension rates relative to non-probainognathians, with an even greater reduction in *Morganucodon*. This would demonstrate an evolutionary plasticity of enamel developmental rates with convergence on reduced enamel extension rates in separate lineages within probainognathians.

#### 4.4.2. Exaeretodon

Plasticity is also shown in the unusual pattern of DSR and CER of *E. riograndensis*. This highly derived species differs not only from other non-probainognathians (i.e. other gomphodontians and the basal epicynodont *Thrinaxodon*), to which it is phylogenetically most closely related, but also from non-hypsodont mammals, mammaliamorphs and hypsodont mammals. It had a CER similar to those of non-hypsodont mammals, but much greater DSR values, which are comparable with those of hypsodont mammals or domesticated dwarf *Sus scrofa* (which are brachydont, but with relatively thick enamel). We suggest that the high DSR of *Exaeretodon* may have been an adaptation to growing a much thicker enamel layer than other non-mammalian cynodonts. Across all other non-mammalian cynodont species, the enamel layer is very thin; maximum enamel thickness ranges from 39 µm in *Morganucodon* to only 125 µm in *Diademodon* (mean = 89 µm; electronic supplementary material, S8). This compares with a maximum enamel thickness of 431 µm in *Exaeretodon*. This is probably an additional adaptation for grinding in its highly specialized and robust triturating dentition [124]. To achieve this greater enamel thickness, a higher DSR would be required, rather than a CER, which contributes to increased crown height in artiodactyls [15] and elephantids [13], but not necessarily to increased enamel thickness.

The reduction in CER in *Exaeretodon* in comparison to other non-probainognathians most likely relates to the fact that it does not replace its postcanine teeth during ontogeny; rather, it loses anterior postcanines and adds new ones at the end of the tooth row in a similar manner to tritylodontids [127,128,137]. It therefore has reduced dental replacement patterns in comparison with other non-probainognathians and, like mammaliamorphs, also shows reduced CER. *Exaeretodon* is therefore consistent with the hypothesis linking reduced CER with reduced dental replacement. From bone histology, its growth appears similar to the flexible growth strategy of other traversodontid cynodonts, although with more rapid juvenile growth [117]. This trend towards increased juvenile bone deposition rates in traversodontids parallels the trend seen from stemward non-mammalian cynodonts to more crownward forms [57,58,117]. As in many non-mammalian cynodonts, growth slowed as the individual matured [117]. It is possible that *Exaeretodon* had achieved a more mammal-like pattern of growth than other traversodontids, with faster juvenile growth and more abrupt termination of growth, as in mammaliaforms, which may also partially explain its reduced CER. It is interesting to note that the mammaliamorph-like pattern of enamel deposition in *Exaeretodon* is consistent with other derived traits of its skull and postcranial anatomy which show similarities with mammaliamorphs [128]; Rowe [30] even positioned *Exaeretodon* as the sister taxon of Mammaliamorpha, although it has since been shown that this taxon is well nested within gomphodonts and such features are therefore mostly convergent with mammaliamorphs [31,138].

## 5. Summary

Periodic increments in the enamel of non-mammalian cynodonts resemble the daily laminations and longer period striae of Retzius of crown mammals. We compared two enamel growth measures, DSR and CER, in crown mammals and diapsids and in two non-mammalian cynodont groups, non-probainognathians and mammaliamorphs. The CER was extremely high in non-probainognathians; enamel extended rapidly in sheath-like layers, in comparison with the gradual accretion of enamel in more crownward mammaliamorphs. This is consistent with reduction in rates of dental replacement in mammaliamorphs, which would permit slower crown extension, while greater replacement rates in non-probainognathians may have required more rapid formation rates. Additionally, low rates of ameloblast differentiation, which cause a low CER, may be coupled with reduced osteoblast function. A low CER in mammaliamorphs therefore suggests reduced rates of bone growth during postcanine formation, consistent with more mammal-like abrupt termination of growth in these animals in comparison with more stemward cynodonts.

## Supplementary Material

Supplementary Material, O'Meara et al. RSOS

## Supplementary Material

Phylogenetic Tree, O'Meara et al. RSOS

## Data accessibility

All primary data and details of statistical analyses are available in the electronic supplementary material.
