# Supplementary material for: Enamel formation and growth in non-mammalian cynodonts
Source: R Soc Open Sci. 2018 May 16;5(5):172293. doi: 10.1098/rsos.172293 (PMC5990740; doi:10.1098/rsos.172293)
Supplement: Supplementary Material, O'Meara et al. RSOS [file rsos172293supp1.docx]

**Supplementary Material**

**Enamel formation and growth in non-mammalian cynodonts**

Rachel N. OʼMeara^1^*, Wendy Dirks^2^ and Agustín G. Martinelli^3,4^

^1^ The University Museum of Zoology, Downing Street, Cambridge, CB2 3EJ

^2^ Department of Anthropology, Durham University, Dawson Building, South Road, Durham, DH1 3LE

^3^ Departamento de Geociências, Universidade Federal do Rio Grande do Sul, Ave. Bento Gonçalves 9500, 91540-000, Porto Alegre, RS, Brazil

^4^ Sección Paleontología de Vertebrados, Museo Argentino de Ciencias Naturales 'Bernardino Rivadavia', Av. Ángel Gallardo 470, Buenos Aires, C1405DJR, Argentina.

*corresponding author: [rno21@cam.ac.uk](mailto:rno21@cam.ac.uk)

**Contents**

S1 Enamel growth rates

S2 Literature sources for enamel growth data

S3 Body mass data

S4 Phylogenetic relationships of taxa

4.1 Topology of the phylogenetic tree

4.2 Construction of the phylogenetic tree and references for divergence times

S5 MANOVA of DSR and CER, 2-day periodicity assumed

5.1 Taxa included

5.2 Group summary statistics

5.3 Summary of MANOVA results

5.4 Univariate ANOVAs

5.5 Kruskal-Wallis Tests

S6 MANOVA of DSR and CER, 1-day periodicity assumed

6.1 Taxa included

6.2 Group summary statistics

6.3 Summary of MANOVA results

6.4 Univariate ANOVAs

S7 Assessing enamel growth rates in *Irajatherium*

7.1 *Irajatherium* included as a mammaliamorph

7.2 *Irajatherium* included as a non-probainognathian

S8 Maximum enamel thickness in non-mammalian cynodonts

S9 References

**S1. Enamel growth rates**

Measures of enamel growth rate in five groups of amniote (diapsids, non-probainognathian cynodonts, mammaliamorphs, non-hypsodont mammals and hypsodont mammals). Average daily secretion rate (DSR) and average crown extension rate (CER) for each species, with mean average DSR and mean average CER for each group. A periodicity of 2-days between long-period lines in the enamel of non-mammalian cynodonts (non-probainognathian cynodont and mammaliamorph groups) is assumed.

| **Group** | **Species** | **Average Daily Secretion Rate (µm/day)** | **Average Crown Extension Rate (µm/day)** | **Group Mean of Average Daily Secretion Rate (µm/day)** | **Group Mean of Average Crown Extension Rate (µm/day)** |
| --- | --- | --- | --- | --- | --- |
| Diapsid | **Crocodylus niloticus* (HT 15/13 A) | 2.29 | 28.38 | 2.60 | 43.5 |
| Diapsid | *†*Borealosuchus wilsoni* (HT 15/13 B) | 2.51 | 72.26 |  |  |
| Diapsid | *Sphenodon punctatus* | 3 | 30 |  |  |
| Non-probainognathian cynodont | *†*Thrinaxodon liorhinus* (UMZC T.814) | 2.24 | 91.12 | 2.71 | 86.0 |
| Non-probainognathian cynodont | *†*Diademodon* sp. (UMZC T.485) | 3.42 | 89.28 |  |  |
| Non-probainognathian cynodont | *†*Diademodon* sp. (UMZC T.480) | 3.31 | 104.09 |  |  |
| Non-probainognathian cynodont | *†*Diademodon* sp. (UMZC T.486) | 2.55 | 103.07 |  |  |
| Non-probainognathian cynodont | *†*Scalenodon angustifrons* (UMZC T.910) | 2.24 | 90.70 |  |  |
| Non-probainognathian cynodont | *†*Massetognathus pascuali* (CRILAR-C 161.1) | 2.67 | 73.80 |  |  |
| Non-probainognathian cynodont | *†*Massetognathus pascuali* (CRILAR-C 161.2) | 2.62 | 73.32 |  |  |
| Non-probainognathian cynodont | *†*Massetognathus pascuali* (CRILAR-C 162.1) | 2.67 | 78.16 |  |  |
| Non-probainognathian cynodont | *†*Massetognathus pascuali* (CRILAR-C 162.2) | 2.66 | 70.34 |  |  |
| Mammaliamorph | *†*Oligokyphus* sp*.* (UMZC T.1236a) | 2.56 | 13.44 | 2.19 | 10.1 |
| Mammaliamorph | *†*Oligokyphus* sp. (UMZC T.1236b) | 2.51 | 15.53 |  |  |
| Mammaliamorph | *†*Oligokyphus* sp. (UMZC T.1236c) | 2.59 | 5.42 |  |  |
| Mammaliamorph | *†*Oligokyphus* sp. (UMZC T.1236d) | 2.31 | 16.80 |  |  |
| Mammaliamorph | *†*Morganucodon watsoni* (UMZC 2017.4.1) | 1.59 | 4.91 |  |  |
| Mammaliamorph | *†*Morganucodon watsoni* (UMZC 2017.4.2) | 1.55 | 4.62 |  |  |
| Non-hypsodont mammal | *Lemur* sp. | 3.6 | 15.3 | 4.11 | 21.8 |
| Non-hypsodont mammal | *Varecia variegatus* | 3.4 | 14.6 |  |  |
| Non-hypsodont mammal | *Galago* sp. | 4.2 | 30.4 |  |  |
| Non-hypsodont mammal | *Nycticebus coucang* | 3.8 | 23.6 |  |  |
| Non-hypsodont mammal | *Perodicticus potto* | 3.2 | 13.8 |  |  |
| Non-hypsodont mammal | *Daubentonia madagascariensis* | 3.7 | 11.5 |  |  |
| Non-hypsodont mammal | *Saguinus midas* | 3.5 | 17.3 |  |  |
| Non-hypsodont mammal | *Ateles* sp. | 3.8 | 9.6 |  |  |
| Non-hypsodont mammal | *Saimiri sciureus* | 4.4 | 21.9 |  |  |
| Non-hypsodont mammal | *Cercopithecus mitis* | 3.6 | 12.6 |  |  |
| Non-hypsodont mammal | *Chlorocebus sabeus* | 3.7 | 11.4 |  |  |
| Non-hypsodont mammal | *Erythrocebus patas* | 4.1 | 15.4 |  |  |
| Non-hypsodont mammal | *Macaca mulatta* | 3.8 | 11.8 |  |  |
| Non-hypsodont mammal | *Papio cynocephalus* | 4.3 | 10.6 |  |  |
| Non-hypsodont mammal | *Pan troglodytes* | 3.8 | 8.9 |  |  |
| Non-hypsodont mammal | *Mus musculus* | 4.0 | 50.0 |  |  |
| Non-hypsodont mammal | *Sus scrofa domesticus* | 20.0 | 87.0 |  |  |
| Non-hypsodont mammal | †*Phenacodus trilobatus* | 3.50 | 18.6 |  |  |
| Non-hypsodont mammal | †*Meniscotherium chamense* | 3.53 | 9.2 |  |  |
| Non-hypsodont mammal | †*Mammuthus columbi* | 3.5 | 47.4 |  |  |
| Non-hypsodont mammal | †*Paleoloxodon cypriotes* | 3.2 | 23.2 |  |  |
| Non-hypsodont mammal | ***†*Ectocion* sp. (WMU-VP 4869) | 4.49 | 20.49 |  |  |
| Non-hypsodont mammal | ***†*Ectocion* sp. (WMU-VP 1349) | 4.43 | 19.85 |  |  |
| Non-hypsodont mammal | **Tupaia tana* (HT 4/09) | 2.74 | 20.91 |  |  |
| Non-hypsodont mammal | **Tupaia tana* (HT 6/09 A) | 2.46 | 21.77 |  |  |
| Non-hypsodont mammal | **Tupaia tana* (HT 6/09 B) | 2.60 | 20.70 |  |  |
| Non-hypsodont mammal | **Tupaia tana* (HT 6/09 C) | 2.58 | 16.59 |  |  |
| Non-hypsodont mammal | **Tupaia tana* (HT 6/09 D) | 2.55 | 17.33 |  |  |
| Non-hypsodont mammal | **Trichechus manatus* (UMZC 2017.3.14) | 3.37 | 15.56 |  |  |
| Non-hypsodont mammal | **Procavia capensis* (HT 11/09 A) | 2.31 | 10.39 |  |  |
| Non-hypsodont mammal | **Procavia capensis* (HT 11/09 B) | 2.46 | 10.83 |  |  |
| Non-hypsodont mammal | **Procavia capensis* (HT 11/09 C) | 2.66 | 9.37 |  |  |
| Non-hypsodont mammal | **Panthera pardus* (HT 66/96 B) | 6.02 | 33.09 |  |  |
| Non-hypsodont mammal | **Canis familiaris* (HT 66/96 A) | 6.53 | 61.64 |  |  |
| Hypsodont mammal | *Ovis aries* (Catalonian) | 11.6 | 150.0 | 12.0 | 113.7 |
| Hypsodont mammal | *Ovis aries* (Soay) | 15.2 | 111.0 |  |  |
| Hypsodont mammal | †*Myotragus balearicu*s | 9.3 | 80.0 |  |  |
| Not grouped for analysis | *†*Exaeretodon riograndensis* (UFRGS-PV-1346-T-S1) | 17.66 | 22.25 | 16.79 | 22.7 |
| Not grouped for analysis | *†*Exaeretodon riograndensis* (UFRGS-PV-1346-T-S2) | 15.92 | 23.16 |  |  |
| Not grouped for analysis | *†*Irajatherium hernandezi* (UFRGS-PV-1348-T-S1) | 1.69 | 44.70 | 1.69 | 44.7 |

* DSR and CER values from specimens sectioned for this study. All other values from literature (see S2 for sources).

† indicates extinct taxa.

**S2. Literature sources for enamel growth data**

Values for daily secretion rate (DSR) and crown extension rate (CER) for teeth of additional crown mammal and crown diapsid species were obtained from the existing literature, and are reported together with their sources in the table below. We included only those species for which both DSR and CER values were available in the same study, and for mammal species we included only data from molar teeth. The relative rarity of studies reporting extension rate data meant that, in order not to limit the sample size of species, we could not restrict the data to a particular molar tooth locus; where the information was available in the original study, we report the locus of the molar from which data was obtained. Where multiple values of DSR for the same species were reported in the original source, in some cases due to use of multiple tooth loci and in others due to measurements being made at different cusps or depths of enamel in the same tooth, the mean was calculated and this is indicated in red. Where a range of crown extension rates from cusp to cervix was reported in the original source, the median of the cuspal and cervical extension rates was used as the average crown extension rate. This is indicated in blue.

| **Taxon** | **Average Daily Secretion Rate (µm/day)** | **Average Crown Extension Rate (µm/day)** | **Tooth locus** | **Reference** |
| --- | --- | --- | --- | --- |
| *Lemur* sp. | 3.6 | 15.3 | M2 | Shellis 1998 |
| *Varecia variegatus* | 3.4 | 14.6 | M1 | Shellis 1998 |
| *Galago* sp. | 4.2 | 30.4 | M1 | Shellis 1998 |
| *Nycticebus coucang* | 3.8 | 23.6 | M1 | Shellis 1998 |
| *Perodicticus potto* | 3.2 | 13.8 | M1 | Shellis 1998 |
| *Daubentonia madagascariensis* | 3.7 | 11.5 | M2 | Shellis 1998 |
| *Saguinus midas* | 3.5 | 17.3 | m1 | Shellis 1998 |
| *Ateles* sp. | 3.8 | 9.6 | M1 | Shellis 1998 |
| *Saimiri sciureus* | 4.4 | 21.9 | m1 | Shellis 1998 |
| *Cercopithecus mitis* | 3.6 | 12.6 | m2 | Shellis 1998 |
| *Chlorocebus sabeus* | 3.7 | 11.4 | m2 | Shellis 1998 |
| *Erythrocebus patas* | 4.1 | 15.4 | m2 | Shellis 1998 |
| *Macaca mulatta* | 3.8 | 11.8 | m1 | Shellis 1998 |
| *Papio cynocephalus* | 4.3 | 10.6 | m1 | Shellis 1998 |
| *Pan troglodytes* | 3.8 | 8.9 | m1 | Shellis 1998 |
| *Mus musculus* | 4.0 | 50.0 | Average across m1, m2 and m3 | Sehic et al. 2013 |
| *Sus scrofa domesticus* | 20.0 | 87.0 | m3 | Kierdorf et al. 2014 |
| *Phenacodus trilobatus*† | 3.50 | 18.6 | Upper molar | Dirks et al. 2009 |
| *Meniscotherium chamense*† | 3.53 | 9.2 | Average CER from median of range of extension rates in upper molar and m1, Average DSR from mean of 4x upper molars and 1x m1 | Dirks et al. 2009 |
| *Mammuthus columbi*† | 3.5 | 47.4 | Molar | Dirks et al. 2012 |
| *Paleoloxodon cypriotes*† | 3.2 | 23.2 | Molar | Dirks et al. 2012 |
| *Ovis aries* (Catalonian) | 11.6 | 150.0 | Average across m1, m2 and m3 | Jordana & Köhler 2011 |
| *Ovis aries* (Soay) | 15.2 | 111.0 | m1 (average from two locations of same tooth) | Kierdorf et al. 2013 |
| *Myotragus balearicu*s† | 9.3 | 80.0 | Average across m1, m2 and m3 | Jordana & Köhler 2011 |
| *Sphenodon punctatus* | 3.0 | 30 | Not differentiated | Kieser et al. 2009 |

†indicates extinct taxa

**S3. Body mass data**

Body mass data for all species is listed below together with sources. For the majority of extant species, body mass data was taken from the Animal Ageing and Longevity Database (AnAge Database: de Magalhaes & Costa 2009). Where only the genus, and not the species, of a particular taxon was known, we took an average body mass of all species of that genus reported in the AnAge database. Where a range of body masses was reported, we took the median body mass across both sexes. For a number of extinct non-mammalian cynodonts, there were no estimated body masses reported in the literature. For these species, body mass was estimated using regression equations of log body mass against log skull length of extant mammals. For small non-mammalian cynodont species (e.g. *Irajatherium*) a regression equation based on extant insectivorous species was used (Luo et al. 2001; Rowe et al. 2011), while for larger species (e.g. *Scalenodon angustifrons*) data from extant mammals with skull lengths ranging between 50 and 170mm were used (Fitch 2000).

| **Taxon** | **Mass (g)** | **Reference** |
| --- | --- | --- |
| *Crocodylus niloticus* | 139784* | Cott 1961 |
| *Borealosuchus wilsoni* | 26400 | Paleobiology Database |
| *Sphenodon punctatus* | 430 | AnAge Database: de Magalhaes & Costa 2009 |
| *Thrinaxodon liorhinus* | 700 | Hurlburt 1996; Rowe et al. 2011 |
| *Diademodon* sp. | 50000 | Jerison 1973; Rowe et al. 2011 |
| *Scalenodon angustifrons* | 8631 | Estimated from regression of weight against skull length of extant mammals using data from Fitch (2000), and *Scalenodon* skull length (= 130mm: Gaetano & Abdala 2015) |
| *Massetognathus pascuali* | 1865 | Quiroga 1980; Rowe et al. 2011 |
| *Exaeretodon* sp. | 46877 | Quiroga 1980; Rowe et al. 2011 |
| *Irajatherium hernandezi* | 401 | Estimated using regression from Luo et al. (2001), and *Irajatherium* skull length (median skull length = 56mm) |
| *Oligokyphus* sp. | 2404 | Estimated from regression of weight against skull length of extant mammals using data from Fitch (2000), and *Oligokyphus* skull length (= 90mm: Kühne 1956) |
| *Morganucodon watsoni* | 51 | Rowe et al. 2011 |
| *Lemur* sp. | 2555 | AnAge Database: de Magalhaes & Costa 2009 |
| *Varecia variegata* | 3670 | AnAge Database: de Magalhaes & Costa 2009 |
| *Galago* sp. | 213.4 | AnAge Database: de Magalhaes & Costa 2009 |
| *Nycticebus coucang* | 890.5 | AnAge Database: de Magalhaes & Costa 2009 |
| *Perodicticus potto* | 1225 | AnAge Database: de Magalhaes & Costa 2009 |
| *Daubentonia madagascariensis* | 2278 | AnAge Database: de Magalhaes & Costa 2009 |
| *Saguinus midas* | 553.8 | AnAge Database: de Magalhaes & Costa 2009 |
| *Ateles* sp. | 6948.5 | AnAge Database: de Magalhaes & Costa 2009 |
| *Saimiri sciureus* | 925 | AnAge Database: de Magalhaes & Costa 2009 |
| *Cercopithecus mitis* | 9000 | AnAge Database: de Magalhaes & Costa 2009 |
| *Chlorocebus sabeus* | 5750 | Skinner & Smithers 1990 |
| *Erythrocebus patas* | 7750 | AnAge Database: de Magalhaes & Costa 2009 |
| *Macaca mulatta* | 8235 | AnAge Database: de Magalhaes & Costa 2009 |
| *Papio cynocephalus* | 18400 | Altmann et al. 1993 |
| *Pan troglodytes*, female | 44984 | AnAge Database: de Magalhaes & Costa 2009 |
| *Mus musculus* | 20.5 | AnAge Database: de Magalhaes & Costa 2009 |
| *Sus scrofa domesticus* (Hanford minpig) | 75000 | Nunoya et al. 2007 |
| *Phenacodus trilobatus* | 54500 | Thewissen 1990 |
| *Meniscotherium chamense* | 9500 | Williamson & Lucas 1992 |
| *Mammuthus columbi* | 7673000 | Shipman 1992 |
| *Paleoloxodon cypriotes* | 200000 | Davies & Lister 2001 |
| *Ectocion* sp. | 6750† | Gingerich 2003 |
| *Tupaia tana* | 164.1 | AnAge Database: de Magalhaes & Costa 2009 |
| *Trichechus manatus* | 401000 | AnAge Database: de Magalhaes & Costa 2009 |
| *Procavia capensis* | 3600 | AnAge Database: de Magalhaes & Costa 2009 |
| *Panthera pardus* | 53750 | AnAge Database: de Magalhaes & Costa 2009 |
| *Canis familiaris* | 40000 | AnAge Database: de Magalhaes & Costa 2009 |
| *Ovis aries* (Catalonian) | 80000 | AnAge Database: de Magalhaes & Costa 2009 |
| *Ovis aries* (Soay) | 23635‡ | Milner et al. 1999 |
| *Myotragus balearicu*s | 60000 | Lalueza-Fox et al. 2002 |

*mean body mass for males and females of breeding age.

†since species of *Ectocion* is unknown, the mean body mass of all *Ectocion* species for which body mass is estimated is used.

‡mean body mass for all males and females.

**S4. Phylogenetic relationships of taxa**

In all the statistical analyses we carried out, enamel growth rates were first adjusted to take account of differences in body mass between species. This was achieved by conducting phylogenetic generalised least squares (PGLS) regressions of log DSR and log CER of all taxa against log body mass. Using the residuals of PGLS regressions controls for non-independence due to shared phylogenetic history during size-correction (Revell 2009). PGLS regressions were carried out in the statistical program R v.3.4.4, using the package *caper* (Orme et al. 2013). PGLS analysis requires a dated phylogenetic tree of all the species in the dataset. The topology of the tree used in our main analysis is described as follows, with branch lengths expressed in millions of years. This is also available as a Nexus file.

"(((Crocodylus:93.4,Borealosuchus:37.6):184.4,Sphenodon:277.8):42.7,(Thrinaxodon:2.917,((Diademodon:2.456,(Scalenodon:5.547,(Massetognathus:4.424,Exaeretodon:14.424):5.122):0.91):2.183,(Irajatherium:12.823,(Oligokyphus:9.921,(Morganucodon:2.48,((Trichechus:66.59,(Procavia:64.83,(Mammuthus:0.9,Palaeoloxodon:5.019):59.03):1.76):38.41,(((((Sus:65.23,(Myotragus:2.712,(OvisSoay:0.0025,OvisCatalonian:0.0025):5.2975):59.93):9.19,(Meniscotherium:13.06,Phenacodus:13.06):5.56):9.19,Ectocion:24.91):1.36,(Panthera:56.64,Canis:56.64):28.33):10.34,((Mus:85.76,Tupaia:85.76):0.57,((((Nycticebus:32.2,Perodicticus:32.2):2.92,Galago:35.12):24.73,(Daubentonia:51.03,(Lemur:22.8,Varecia:22.8):28.23):8.82):16.05,((Ateles:19.82,(Saguinus:17.37,Saimiri:17.37):2.45):18.53,(Pan:27.48,((Papio:7.08,Macaca:7.08):2.93,(Cercopithecus:7.16,(Chlorocebus:4.31,Erythrocebus:4.31):2.85):2.85):17.47):10.37):38.05):10.43):8.98):9.69):172.07):7.44):8.901):23.817):4.278):65.58);"

**S4.1 Topology of the phylogenetic tree**

**S4.2 Construction of the phylogenetic tree and references for divergence times**

The phylogenetic framework was constructed in Mesquite v.3.04 following the method of Koyabu et al. (2014) in constructing a composite topology with estimated divergence times from multiple phylogenies.

The TimeTree of Life project (Hedges et al. 2015) provides the broad framework of the phylogeny, and estimates of minimum divergence times for major amniote groups based on collated molecular evidence from multiple sources. Within major subgroups, at lower taxonomic levels, estimated divergence times of taxa, and terminal branch lengths were provided by large-scale studies (e.g. Meredith et al. 2011 for crown mammals; Perelman et al. 2011 for primates; Ruta et al. 2013 for non-mammalian cynodonts). Since divergence times from these studies and the *TimeTree of Life* were not always consistent, the divergence times at lower taxonomic levels were adjusted relative to the major clade divergences of the *TimeTree of Life*. At nodes representing the origins of major subgroups, the ratio between the *TimeTree of Life* divergence estimate and the estimate from the more specific study of the subgroup was calculated. This ratio was then used to scale the divergence estimates of the subclades within this node. This produced a composite topology with divergence estimates scaled relative to the broad *TimeTree of Life* framework, so as to be internally consistent. Branch lengths were calculated from these divergence times and are expressed in millions of years. Where a reference was not available for the age of a particular node, the branch lengths of terminal taxa and their containing subclades, or between two nodes of known divergence time, were evenly distributed.

References for the divergence times in the composite topology are presented in the table below. Age 1 = Diveregence time inferred from *TimeTree of Life*. Reference = Literature reference for age of subclade. Age 2 = Divergence time inferred from Reference. At nodes where Age 1 and Age 2 overlapped (indicated by *), a factor of the two was calculated (Age 1/Age2). This factor was then used to scale the ages of the subclades within this node (Age 3).

| **Node** | **Taxon** | **Age 1 (Ma)** | **Reference** | **Age 2 (Ma)** | **Factor** | **Age 3 (Ma)** |
| --- | --- | --- | --- | --- | --- | --- |
| 1 | Amniota | 320.5 | - | - | - | 320.5 |
| 2 | Sauropsida | 277.8 | - | - | - | 277.8 |
| 3 | *Crocodylu*s sp. + *Borealosuchus wilsoni* | min 93.4† | - | - | - | 93.4 |
| 4 | Epicynodontia | - | Ruta et al. 2013 | 254.92 | - | 254.92 |
| 5 | Cynognathia + Probainognathia | - | Ruta et al. 2013 | 250.64 | - | 250.64 |
| 6 | Cynognathia | - | Ruta et al. 2013 | 248.46 | - | 248.46 |
| 7 | *Scalenodon angustifrons* + (*Massetognathus* + *Exaeretodon*) | - | Ruta et al. 2013 | 247.55 | - | 247.55 |
| 8 | *Massetognathus* + *Exaeretodon* | - | Ruta et al. 2013 | 242.42 | - | 242.42 |
| 9 | Probainognathia | - | Ruta et al. 2013 | 226.82 |  | 226.82 |
| 10 | *Oligokyphus* + Mammaliaformes | - | Ruta et al. 2013 | 217.92 | - | 217.92 |
| 11 | Mammaliaformes | - | Ruta et al. 2013 | 210.48 | - | 210.48 |
| 12 | Placentalia | 105.0* | Meredith et al. 2011 | 101.35 | 1.04 | 105.00 |
| 13 | Paenungulata | - | Meredith et al. 2011 | 64.28 | - | 66.59 |
| 14 | Proboscidea + Hyracoidea | - | Meredith et al. 2011 | 62.58 | - | 64.83 |
| 15 | *Mammuthus columbi* + *Elephas cypriotes* | - | Poulakakis et al. 2006§ | 5.8 | - | 5.8 |
| 16 | Boreoeutheria | - | Meredith et al. 2011 | 92.00 | - | 95.31 |
| 17 | Variamana (Chiroptera + Perissodactyla + Pholidota + Carnivora + Cetartiodactyla) | - | Meredith et al. 2011 | 82.02 | - | 84.97 |
| 18 | Carnivora | - | Meredith et al. 2011 | 54.67 | - | 56.64 |
| 19 | Euungulata (Perissodactyla + Cetartiodactyla) | - | Meredith et al. 2011, FAD used to calculate branch length of *Ectocion*¶ | 80.70 | - | 83.61 |
| 20 | Artiodactyla + *Phenacodus trilobatus* + *Meniscotherium chamense* | - | Internal branch lengths (between nodes 21 and 22, and 22 and 24) evenly distributed | - | - | 74.42 |
| 21 | *Phenacodus trilobatus* + *Meniscotherium chamense* | - | FADs used to calculate branch lengths¶ | - | - | 68.86 |
| 22 | Ruminantia + Whippomorpha + Suinamorpha | - | Meredith et al. 2011 | 62.96 | - | 65.23 |
| 23 | *Myotragus balearicus* + *Ovis aries* | - | Brown & Yang 2010 | 5.3 | - | 5.3 |
| 24 | *Ovis aries* (Catalonian breed + Soay breed) | - | Kijas et al. 2012 | 0.0025 | - | 0.0025 |
| 25 | Euarchontoglires | - | Meredith et al. 2011 | 83.33 | - | 86.33 |
| 26 | Scandentia + Glires | - | Meredith et al. 2011 | 82.78 | - | 85.76 |
| 27 | Primata | 75.9* | Perelman et al. 2011 | 87.18 | 0.87 | 75.90 |
| 28 | Strepsirhini | - | Perelman et al. 2011 | 68.74 | - | 59.85 |
| 29 | Lemuriformes | - | Perelman et al. 2011 | 58.61 | - | 51.03 |
| 30 | Lemuridae (*Lemur* sp. + *Varencia variegatus*) | - | Perelman et al. 2011 | 26.19 | - | 22.80 |
| 31 | Lorisiformes | - | Perelman et al. 2011 | 40.34 | - | 35.12 |
| 32 | Lorisidae (*Nycticebus coucang* + *Perodicticus* *potto*) | - | Perelman et al. 2011 | 36.98 | - | 32.20 |
| 33 | Haplorhini | - | Perelman et al. 2011 | 43.47 | - | 37.85 |
| 34 | Platyrhini | - | Perelman et al. 2011 | 22.76 | - | 19.82 |
| 35 | Cebidae (*Saguinus midas* + *Saimiri sciureus*) | - | Perelman et al. 2011 | 19.95 | - | 17.37 |
| 36 | Catarrhini | - | Perelman et al. 2011 | 31.56 | - | 27.48 |
| 37 | Cercopithecidae | - | Perelman et al. 2011 | 11.50 | - | 10.01 |
| 38 | Papionini (*Macaca mulatta* + *Papio cynocephalus*) | - | Perelman et al. 2011 | 8.13 | - | 7.08 |
| 39 | Cercopithecini (*Cercopithecus mitis* + (*Erythrocebus patas* + *Chlorocebus sabaeus*)) | - | Perelman et al. 2011 | 8.22 | - | 7.16 |
| 40 | *Erythrocebus patas* + *Chlorocebus sabaeus* | - | Perelman et al. 2011 | 4.95 | - | 4.31 |

† *Alligator*-*Crocodylus* divergence date from *TimeTree of Life* used as minimum estimate since it certainly occurred after *Borealosuchus*-*Crocodylus* divergence.

± *Irajatherium* was added to the phylogeny of Ruta et al. (2013); it is nested within Ictidosauria as the sister group to all other tritheledontids, following (Oliviera et al. 2011).

§ *Mammuthus*-*Elephas* divergence time from *TimeTree of Life* was used for this clade since *Palaeoloxodon cyriotes* is recovered within the *Elephas* clade (Poulakakis et al. 2006).

¶ *Ectocion* is resolved as a stem-perrisodactyl in Halliday et al. (2017), with *Phenacodus* and *Meniscotherium* as stem-artiodactyls. The FAD of *Ectocion* (58.7Ma) was used to estimate its terminal branch length. The FAD of *Phenacodus* (55.8 Ma) was used to estimate divergence time at node 23 by evenly distributing branch lengths between the terminal taxon *Phenacodus* and its containing containing subclade. FADs of *Phenacodus trilobatus* (55.8 Ma) and *Meniscotherium chamense* (55.8 Ma) were then used to calculate terminal branch lengths for these taxa.

Where terminal branch lengths were not available from large-scale dated phylogenetic studies, these were calculated for each taxon using its First Appearance Datum (FAD). These FADS are listed below; all are from the Palaebiology Database (REF) with the exception of that of *Irajatherium*, which is from Ruta et al. (2013).

| **Taxon** | | | **FAD (Ma)** |
| --- | --- | --- | --- |
| *Borealosuchus wilsoni* | | | 55.8 |
| *Irajatherium hernandezi* | | | 214 |
| *Mammuthus columbi* | | | 4.9 |
| *Palaeoloxodon (Elephas) cypriotes* | | 0.781 | |
| *Phenacodus trilobatus* | 55.8 | | |
| *Meniscotherium chamense* | 55.8 | | |
| *Ectocion* | 58.7 | | |
| *Myotragus balearicus* | 2.588 | | |

**S5. MANOVA of DSR and CER, 2-day periodicity assumed.**

Multivariate analysis of variance (Tabachnick & Fidell 2007) was carried out in the statistical program R v.3.4.4 to assess differences between five groups across two dependent variables. The two dependent variables related to the growth rate of enamel: the daily secretion rate (DSR) and crown extension rate (CER). These growth rates were adjusted to take account of differences in body mass between species, while controlling for non-independence due to phylogenetic history, by performing phylogenetic generalised least squares regressions (Revell 2009) of the log DSR and log CER of all taxa against log body mass. We then used the residuals from these regressions as body mass corrected DSR and body mass corrected CER. These variables were also Box-Cox transformed for multivariate normality (Sokal & Rohlf 2012). The five groups were diapsids, non-probainognathians, mammaliamorphs, non-hypsodont crown mammals, and hypsodont crown mammals.

For the two non-mammalian cynodont groups (non-probainognathians and mammaliamorphs) average DSR and average CER for each species were used as individual data points. A total of 39 taxa were included in the analysis. DSR and CER were calculated assuming a 2-day periodicity between long-period lines in non-mammalian cynodonts. Post-hoc pairwise comparisons between the different groups were carried out, and p-values were adjusted for multiple comparisons using false discovery rate (FDR) correction (Benjamini & Hochberg 1995; Curran-Everett 2000). Separate univariate ANOVAs were carried out on each enamel growth rate dependent variable, and for each variable pairwise comparisons of each group were performed.

**S5.1. Taxa included:**

Diapsid: *Crocodylus niloticus, Borealosuchus wilsoni* and *Sphenodon punctatus.*

Non-probainognathian cynodont: *Thrinaxodon liorhinus, Diademodon* sp., *Scalenodon angustifrons* and *Massetognathus pascuali*.

Mammaliamorph: *Oligokyphus* sp. and *Morganucodon watsoni.*

Non-hypsodont crown mammal: *Lemur* sp., *Varecia variegatus, Galago* sp., *Nycticebus coucang, Perodicticus potto, Daubentonia madagascariensis, Saguinus midas, Ateles* sp., *Saimiri sciureus, Cercopithecus mitis, Chlorocebus sabeus, Erythrocebus patas, Macaca mulatta, Papio cynocephalus, Pan troglodytes, Mus musculus, Sus scrofa domesticus, Phenacodus trilobatus, Mensicotherium chamense, Mammuthus columbi, Paleoloxodon cypriotes, Ectocion* sp., *Tupaia tana*, *Trichechus manatus, Procavia capensis, Panthera pardus* and *Canis familiaris.*

Hypsodont crown mammal: *Ovis aries* (Catalonian breed), *Ovis aries* (Soay breed) and *Myotragus balearicus.*

**S5.2. Group summary statistics**

The original means and standard deviations of DSR and CER for each group are shown in the table below, together with means and standard deviations of these variables following body mass correction (in a phylogenetic context), and Box-Cox transformation.

| **Enamel Growth Variable** | **Group** | **Mean of Original Variable (µm/day)** | **Std. Deviation of Original Variable (µm/day)** | **Mean of Variable following Body Mass Correction (in a phylogenetic context) and Transformation** | **Std. Deviation of Variable following Body Mass Correction (in a phylogenetic context) and Transformation** |
| --- | --- | --- | --- | --- | --- |
| DSR | Diapsid | 2.60 | 0.36 | 0.9017 | 0.1066 |
|  | Non-Probainognathian | 2.56 | 0.41 | 0.8798 | 0.0406 |
|  | Mammaliamorph | 2.03 | 0.65 | 0.9147 | 0.0635 |
|  | Non-Hypsodont Mammal | 4.45 | 3.22 | 0.7700 | 0.0711 |
|  | Hypsodont Mammal | 12.03 | 2.97 | 0.6098 | 0.0307 |
| CER | Diapsid | 43.55 | 24.88 | 1.0419 | 0.0106 |
|  | Non-Probainognathian | 88.64 | 10.50 | 1.0558 | 0.0026 |
|  | Mammaliamorph | 8.79 | 5.68 | 1.0180 | 0.0091 |
|  | Non-Hypsodont Mammal | 23.12 | 18.46 | 1.0276 | 0.0141 |
|  | Hypsodont Mammal | 113.66 | 35.08 | 1.0545 | 0.0040 |

**Plot of DSR vs. CER**

The DSR and CER of the taxa included in this MANOVA are illustrated in the figure below. A) Raw growth rates: daily secretion rate (µm/day) vs. crown extension rate (µm/day). B) Body mass corrected growth rates: the residuals of log daily secretion rate (µm/day) and log crown extension rate (µm/day) following phylogenetic generalised least squares regression against log body mass.

**S5.3. Summary of MANOVA results**

Multivariate analysis of variance comparing two enamel growth rate variables (DSR and CER, both body mass corrected in a phylogenetic context, and transformed) across five different groups

**Wilks’ Lambda, significance and effect sizes**

| **Effect** | **Wilks' Lambda** | **F-value** | **Degrees of freedom** | **p-value** | **Partial η2  (effect size)** |
| --- | --- | --- | --- | --- | --- |
| Group | 0.169 | 11.834 | 8,66 | 0.00000000 | 0.589 |

**Significance of pairwise comparisons**

The significances of pairwise comparisons between groups are adjusted for multiple comparisons by FDR correction. P-values which indicate significant differences between groups at α < 0.05 are highlighted in red.

|  | **Diapsid** | **Non-Probainognathian** | **Mammaliamorph** | **Non-Hypsodont Mammal** |
| --- | --- | --- | --- | --- |
| **Non-Probainognathian** | 0.1574 | - | - | - |
| **Mammaliamorph** | 0.1944 | 0.0163 | - | - |
| **Non-Hypsodont Mammal** | 0.0006 | 0.0000 | 0.0448 | - |
| **Hypsodont Mammal** | 0.0732 | 0.0059 | 0.0713 | 0.0059 |

**S5.4. Univariate ANOVAS**

Since the two dependent variables (DSR and CER, body mass corrected in a phylogenetic context and transformed) were relatively uncorrelated (pooled within-group correlation = 0.428), it was appropriate to carry out separate univariate ANOVAs each of these variables (Tabachnick & Fidell 2007).

| **Dependent Variable  (body mass corrected and transformed)** | **Sum of Squares** | **Mean Squares** | **Degrees of freedom** | **F-value** | **p-value (FDR adjusted)** | **Partial η^2^ (effect size)** |
| --- | --- | --- | --- | --- | --- | --- |
| DSR | 0.209 | 0.052 | 4,34 | 10.75 | 0.00001938 | 0.558 |
| CER | 0.005 | 0.001 | 4,34 | 7.578 | 0.00017700 | 0.471 |

For each variable pairwise comparisons of each group were performed and significances were corrected using FDR. P-values which indicate significant differences between groups at α < 0.05 are highlighted in red.

**DSR** (body mass corrected in a phylogenetic context, and transformed)

|  | **Diapsid** | **Non-Probainognathian** | **Mammaliamorph** | **Non-Hypsodont Mammal** |
| --- | --- | --- | --- | --- |
| **Non-Probainognathian** | 0.7526 | - | - | - |
| **Mammaliamorph** | 0.8900 | 0.5200 | - | - |
| **Non-Hypsodont Mammal** | 0.0168 | 0.0162 | 0.0189 | - |
| **Hypsodont Mammal** | 0.0189 | 0.0042 | 0.0162 | 0.0046 |

**CER** (body mass corrected in a phylogenetic context, and transformed)

|  | **Diapsid** | **Non-Probainognathian** | **Mammaliamorph** | **Non-Hypsodont Mammal** |
| --- | --- | --- | --- | --- |
| **Non-Probainognathian** | 0.0800 | - | - | - |
| **Mammaliamorph** | 0.1255 | 0.0051 | - | - |
| **Non-Hypsodont Mammal** | 0.1471 | 0.0046 | 0.4438 | - |
| **Hypsodont Mammal** | 0.1667 | 0.7078 | 0.0168 | 0.0122 |

**S5.5. Kruskal-Wallis tests**

Kruskal-Wallis tests, non-parametric versions of the univariate ANOVAs, were carried out on the two dependent variables (DSR and CER, corrected for body mass in a phylogenetic context).

| **Dependent Variable  (body mass corrected)** | **Kruskal-Wallis χ^2^** | **Degrees of freedom** | **p-value (FDR adjusted)** |
| --- | --- | --- | --- |
| DSR | 18.976 | 4 | 0.00167 |
| CER | 17.330 | 4 | 0.00159 |

For each variable pairwise Dunn tests were performed and significances were corrected using FDR.
P-values which indicate significant differences between groups at α < 0.05 are highlighted in red.

**DSR** (body mass corrected in a phylogenetic context)

|  | **Diapsid** | **Non-Probainognathian** | **Mammaliamorph** | **Non-Hypsodont Mammal** |
| --- | --- | --- | --- | --- |
| **Non-Probainognathian** | 0.4847 | - | - | - |
| **Mammaliamorph** | 0.4639 | 0.4639 | - | - |
| **Non-Hypsodont Mammal** | 0.0346 | 0.0238 | 0.0400 | - |
| **Hypsodont Mammal** | 0.0060 | 0.0060 | 0.0060 | 0.0346 |

**CER** (body mass corrected in a phylogenetic context)

|  | **Diapsid** | **Non-Probainognathian** | **Mammaliamorph** | **Non-Hypsodont Mammal** |
| --- | --- | --- | --- | --- |
| **Non-Probainognathian** | 0.2059 | - | - | - |
| **Mammaliamorph** | 0.0942 | 0.0153 | - | - |
| **Non-Hypsodont Mammal** | 0.1234 | 0.0060 | 0.2616 | - |
| **Hypsodont Mammal** | 0.2472 | 0.4822 | 0.0238 | 0.0153 |

**S6. MANOVA of DSR and CER, 1-day periodicity assumed.**

Multivariate analysis of variance (Tabachnick & Fidell 2007) was carried out in the statistical program R v.3.4.4 to assess differences between five groups across two dependent variables. The two dependent variables related to the growth rate of enamel: the daily secretion rate (DSR) and crown extension rate (CER). These growth rates were adjusted to take account of differences in body mass between species, while controlling for non-independence due to phylogenetic history, by performing phylogenetic generalised least squares regressions (Revell 2009) of DSR and CER of all taxa against log body mass. We then used the residuals from these regressions as body mass corrected DSR and body mass corrected CER. These variables were also Box-Cox transformed for multivariate normality (Sokal & Rohlf 2012). The five groups were diapsids, non-probainognathians, mammaliamorphs, non-hypsodont crown mammals, and hypsodont crown mammals.

For the two non-mammalian cynodont groups (non-probainognathians and mammaliamorphs) average DSR and average CER for each species were used as individual data points. A total of 39 taxa were included in the analysis. DSR and CER were calculated assuming a 1-day periodicity between long-period lines in non-mammalian cynodonts. Post-hoc pairwise comparisons between the different groups were carried out, and p-values were adjusted for multiple comparisons using false discovery rate (FDR) correction (Benjamini & Hochberg 1995; Curran-Everett 2000). Separate univariate ANOVAs were carried out on each enamel growth rate dependent variable, and for each variable pairwise comparisons of each group were performed.

**S6.1. Taxa included:**

Diapsid: *Crocodylus niloticus, Borealosuchus wilsoni* and *Sphenodon punctatus.*

Non-probainognathian cynodont: *Thrinaxodon liorhinus, Diademodon* sp., *Scalenodon angustifrons* and *Massetognathus pascuali*.

Mammaliamorph: *Oligokyphus* sp. and *Morganucodon watsoni.*

Non-hypsodont crown mammal: *Lemur* sp., *Varecia variegatus, Galago* sp., *Nycticebus coucang, Perodicticus potto, Daubentonia madagascariensis, Saguinus midas, Ateles* sp., *Saimiri sciureus, Cercopithecus mitis, Chlorocebus sabeus, Erythrocebus patas, Macaca mulatta, Papio cynocephalus, Pan troglodytes, Mus musculus, Sus scrofa domesticus, Phenacodus trilobatus, Mensicotherium chamense, Mammuthus columbi, Paleoloxodon cypriotes, Ectocion* sp., *Tupaia tana*, *Trichechus manatus, Procavia capensis, Panthera pardus* and *Canis familiaris.*

Hypsodont crown mammal: *Ovis aries* (Catalonian breed), *Ovis aries* (Soay breed) and *Myotragus balearicus.*

**S6.2. Group summary statistics**

The original means and standard deviations of DSR and CER for each group are shown in the table below, together with means and standard deviations of these variables following body mass correction (in a phylogenetic context), and Box-Cox transformation.

| **Enamel Growth Variable** | **Group** | **Mean of Original Variable (µm/day)** | **Std. Deviation of Original Variable (µm/day)** | **Mean of Variable following Body Mass Correction (in a phylogenetic context) and Transformation** | **Std. Deviation of Variable following Body Mass Correction (in a phylogenetic context) and Transformation** |
| --- | --- | --- | --- | --- | --- |
| DSR | Diapsid | 2.60 | 0.36 | 0.9353 | 0.0632 |
|  | Non-Probainognathian | 4.88 | 0.86 | 0.8447 | 0.0103 |
|  | Mammaliamorph | 3.92 | 1.34 | 0.8476 | 0.0102 |
|  | Non-Hypsodont Mammal | 4.45 | 3.22 | 0.8711 | 0.0471 |
|  | Hypsodont Mammal | 12.03 | 2.97 | 0.7699 | 0.0236 |
| CER | Diapsid | 43.55 | 24.88 | 2.1490 | 0.2831 |
|  | Non-Probainognathian | 177.39 | 22.90 | 2.8293 | 0.0569 |
|  | Mammaliamorph | 16.88 | 11.12 | 2.1706 | 0.0644 |
|  | Non-Hypsodont Mammal | 23.12 | 18.46 | 1.9564 | 0.2981 |
|  | Hypsodont Mammal | 113.66 | 35.08 | 2.5363 | 0.1569 |

**Plot of DSR vs. CER**

The DSR and CER of the taxa included in this MANOVA are illustrated in the figure below. A) Raw growth rates: daily secretion rate (µm/day) vs. crown extension rate (µm/day). B) Body mass corrected growth rates: the residuals of daily secretion rate (µm/day) and crown extension rate (µm/day) following phylogenetic generalised least squares regression against log body mass.

**S6.3. Summary of MANOVA results**

Multivariate analysis of variance comparing two enamel growth rate variables (DSR and CER, both body mass corrected in a phylogenetic context, and transformed) across five different groups.

**Wilks’ Lambda, significance and effect sizes**

| **Effect** | **Wilks' Lambda** | **F-value** | **Degrees of freedom** | **p-value** | **Partial η2  (effect size)** |
| --- | --- | --- | --- | --- | --- |
| Group | 0.205 | 9.989 | 8,66 | 0.00000001 | 0.548 |

**Significance of pairwise comparisons**

The significances of pairwise comparisons between groups are adjusted for multiple comparisons by FDR correction. P-values which indicate significant differences between groups at α < 0.05 are highlighted in red.

|  | **Diapsid** | **Non-Probainognathian** | **Mammaliamorph** | **Non-Hypsodont Mammal** |
| --- | --- | --- | --- | --- |
| **Non-Probainognathian** | 0.1574 | - | - | - |
| **Mammaliamorph** | 0.1944 | 0.0163 | - | - |
| **Non-Hypsodont Mammal** | 0.0006 | 0.0000 | 0.0448 | - |
| **Hypsodont Mammal** | 0.0732 | 0.0059 | 0.0713 | 0.0059 |

**S6.4. Univariate ANOVAS**

Since the two dependent variables (DSR and CER, body mass corrected in a phylogenetic context and transformed) were relatively uncorrelated (pooled within-group correlation = 0.562), it was appropriate to carry out separate univariate ANOVAs each of these variables (Tabachnick & Fidell 2007).

| **Dependent Variable  (body mass corrected and transformed)** | **Sum of Squares** | **Mean Squares** | **Degrees of freedom** | **F-value** | **p-value (FDR adjusted)** | **Partial η^2^ (effect size)** |
| --- | --- | --- | --- | --- | --- | --- |
| DSR | 0.045 | 0.0113 | 4,34 | 5.725 | 0.00124000 | 0.402 |
| CER | 3.262 | 0.8154 | 4,34 | 10.94 | 0.00001644 | 0.563 |

For each variable pairwise comparisons of each group were performed and significances were corrected using FDR. P-values which indicate significant differences between groups at α < 0.05 are highlighted in red.

**DSR** (body mass corrected in a phylogenetic context, and transformed)

|  | **Diapsid** | **Non-Probainognathian** | **Mammaliamorph** | **Non-Hypsodont Mammal** |
| --- | --- | --- | --- | --- |
| **Non-Probainognathian** | 0.0666 | - | - | - |
| **Mammaliamorph** | 0.2300 | 0.8021 | - | - |
| **Non-Hypsodont Mammal** | 0.0685 | 0.3700 | 0.5489 | - |
| **Hypsodont Mammal** | 0.0377 | 0.0109 | 0.0243 | 0.0075 |

**CER** (body mass corrected in a phylogenetic context, and transformed)

|  | **Diapsid** | **Non-Probainognathian** | **Mammaliamorph** | **Non-Hypsodont Mammal** |
| --- | --- | --- | --- | --- |
| **Non-Probainognathian** | 0.0158 | - | - | - |
| **Mammaliamorph** | 0.9260 | 0.0021 | - | - |
| **Non-Hypsodont Mammal** | 0.3700 | 0.0001 | 0.3847 | - |
| **Hypsodont Mammal** | 0.1646 | 0.0418 | 0.0475 | 0.0110 |

**S7. Assessing enamel growth rates in *Irajatherium***

MANOVAs were carried out as in section S5 (assuming a 2-day periodicity of the long-period lines) but including the tritheledontid *Irajatherium*. *Irajatherium* was included either in the mammaliamorph or in the non-probainognathian group in order to evaluate its intermediate position between these two groups.

**Plot of DSR vs. CER**

The position of *Irajatherium* is indicated in the DSR vs. CER plot below. A) Raw growth rates: daily secretion rate (µm/day) vs. crown extension rate (µm/day). B) Body mass corrected growth rates: the residuals of log daily secretion rate (µm/day) and log crown extension rate (µm/day) following phylogenetic generalised least squares regression against log body mass.

**S7.1.1 *Irajatherium* included as a mammaliamorph**

Multivariate analysis of variance comparing two enamel growth rate variables (DSR and CER, both body mass corrected in a phylogenetic context, and transformed) across five different groups. *Irajatherium* was included in the mammaliamorph group.

**Wilks’ Lambda, significance and effect sizes**

| **Effect** | **Wilks' Lambda** | **F-value** | **Degrees of freedom** | **p-value** | **Partial η^2^ (effect size)** |
| --- | --- | --- | --- | --- | --- |
| Group | 0.190 | 11.025 | 8,68 | 0.00000000 | 0.565 |

**Significance of pairwise comparisons**

The significances of pairwise comparisons between groups are adjusted for multiple comparisons by FDR correction. P-values which indicate significant differences between groups at α < 0.05 are highlighted in red.

|  | **Diapsid** | **Non-Probainognathian** | **Mammaliamorph** | **Non-Hypsodont Mammal** |
| --- | --- | --- | --- | --- |
| **Non-Probainognathian** | 0.1516 | - | - | - |
| **Mammaliamorph** | 0.6943 | 0.0358 | - | - |
| **Non-Hypsodont Mammal** | 0.0007 | 0.0000 | 0.0024 | - |
| **Hypsodont Mammal** | 0.0868 | 0.0046 | 0.0133 | 0.0046 |

**S7.1.2 Univariate ANOVAS**

Separate univariate ANOVAs were carried out on each of these variables (Tabachnick & Fidell 2007).

| **Dependent Variable  (body mass corrected and transformed)** | **Sum of Squares** | **Mean Squares** | **Degrees of freedom** | **F-value** | **p-value (FDR adjusted)** | **Partial η^2^ (effect size)** |
| --- | --- | --- | --- | --- | --- | --- |
| DSR | 0.2014 | 0.0503 | 4,34 | 12.28 | 0.00000486 | 0.584 |
| CER | 0.0340 | 0.0085 | 4,34 | 6.536 | 0.00048800 | 0.428 |

For each variable pairwise comparisons of each group were performed and significances were corrected using FDR. P-values which indicate significant differences between groups at α < 0.05 are highlighted in red.

**DSR** (body mass corrected in a phylogenetic context, and transformed)

|  | **Diapsid** | **Non-Probainognathian** | **Mammaliamorph** | **Non-Hypsodont Mammal** |
| --- | --- | --- | --- | --- |
| **Non-Probainognathian** | 0.7495 | - | - | - |
| **Mammaliamorph** | 0.7495 | 0.2613 | - | - |
| **Non-Hypsodont Mammal** | 0.0193 | 0.0187 | 0.0039 | - |
| **Hypsodont Mammal** | 0.0231 | 0.0039 | 0.0039 | 0.0039 |

**CER** (body mass corrected in a phylogenetic context, and transformed)

|  | **Diapsid** | **Non-Probainognathian** | **Mammaliamorph** | **Non-Hypsodont Mammal** |
| --- | --- | --- | --- | --- |
| **Non-Probainognathian** | 0.0811 | - | - | - |
| **Mammaliamorph** | 0.4763 | 0.0446 | - | - |
| **Non-Hypsodont Mammal** | 0.1512 | 0.0039 | 0.9030 | - |
| **Hypsodont Mammal** | 0.1657 | 0.7495 | 0.1512 | 0.0082 |

**S7.2.1 *Irajatherium* included as a non-probainognathian**

Multivariate analysis of variance comparing two enamel growth rate variables (DSR and CER, both body mass corrected in a phylogenetic context, and transformed) across five different groups. *Irajatherium* was included in the non-probainognathian group.

**Wilks’ Lambda, significance and effect sizes**

| **Effect** | **Wilks' Lambda** | **F-value** | **Degrees of freedom** | **p-value** | **Partial η^2^ (effect size)** |
| --- | --- | --- | --- | --- | --- |
| Group | 0.150 | 13.453 | 8,68 | 0.00000000 | 0.613 |

**Significance of pairwise comparisons**

The significances of pairwise comparisons between groups are adjusted for multiple comparisons by FDR correction. P-values which indicate significant differences between groups at α < 0.05 are highlighted in red.

|  | **Diapsid** | **Non-Probainognathian** | **Mammaliamorph** | **Non-Hypsodont Mammal** |
| --- | --- | --- | --- | --- |
| **Non-Probainognathian** | 0.0817 | - | - | - |
| **Mammaliamorph** | 0.1946 | 0.0037 | - | - |
| **Non-Hypsodont Mammal** | 0.0007 | 0.0000 | 0.0602 | - |
| **Hypsodont Mammal** | 0.0760 | 0.0035 | 0.0682 | 0.0042 |

**S7.2.2 Univariate ANOVAS**

Separate univariate ANOVAs were carried out on each of these variables (Tabachnick & Fidell 2007).

| **Dependent Variable  (body mass corrected and transformed)** | **Sum of Squares** | **Mean Squares** | **Degrees of freedom** | **F-value** | **p-value (FDR adjusted)** | **Partial η^2^ (effect size)** |
| --- | --- | --- | --- | --- | --- | --- |
| DSR | 0.1974 | 0.0494 | 4,35 | 11.71 | 0.00000772 | 0.527 |
| CER | 0.0395 | 0.0099 | 4,35 | 8.65 | 0.00005780 | 0.497 |

For each variable pairwise comparisons of each group were performed and significances were corrected using FDR. P-values which indicate significant differences between groups at α < 0.05 are highlighted in red.

**DSR** (body mass corrected in a phylogenetic context, and transformed)

|  | **Diapsid** | **Non-Probainognathian** | **Mammaliamorph** | **Non-Hypsodont Mammal** |
| --- | --- | --- | --- | --- |
| **Non-Probainognathian** | 0.9620 | - | - | - |
| **Mammaliamorph** | 0.9620 | 0.9365 | - | - |
| **Non-Hypsodont Mammal** | 0.0175 | 0.0029 | 0.0218 | - |
| **Hypsodont Mammal** | 0.0208 | 0.0019 | 0.0132 | 0.0029 |

**CER** (body mass corrected in a phylogenetic context, and transformed)

|  | **Diapsid** | **Non-Probainognathian** | **Mammaliamorph** | **Non-Hypsodont Mammal** |
| --- | --- | --- | --- | --- |
| **Non-Probainognathian** | 0.0552 | - | - | - |
| **Mammaliamorph** | 0.1200 | 0.0020 | - | - |
| **Non-Hypsodont Mammal** | 0.1403 | 0.0019 | 0.4000 | - |
| **Hypsodont Mammal** | 0.1547 | 0.9620 | 0.0175 | 0.0082 |

**S8. Maximum enamel thickness in non-mammalian cynodonts**

Thickness was measured perpendicular to the enamel dentine junction (EDJ), and was only measured in unworn portions of enamel. In those species for which multiple tooth specimens were available, the mean maximum enamel thickness was calculated.

| **Specimen** | **Maximum enamel thickness (µm)** | **Species mean maximum enamel thickness (µm)** |
| --- | --- | --- |
| *Thrinaxodon liorhinus* (UMZC T.814) | 50.2 | 50.2 |
| *Diademodon* sp. (UMZC T.480) | 109.3 | 124.6 |
| *Diademodon* sp. (UMZC T.485) | 133.6 |  |
| *Diademodon* sp. (UMZC T.486) | 130.8 |  |
| *Scalenodon angustifrons* (UMZC T.910) | 110.0 | 110.0 |
| *Massetognathus pascuali* (CRILAR-C 161.1) | 123.5 | 109.6 |
| *Massetognathus pascuali* (CRILAR-C 161.2) | 115.7 |  |
| *Massetognathus pascuali* (CRILAR-C 162.1) | 92.4 |  |
| *Massetognathus pascuali* (CRILAR-C 162.2) | 106.9 |  |
| *Exaeretodon riograndensis* (UFRGS-PV-1346-T-S1) | 392.9 | 431.3 |
| *Exaeretodon riograndensis* (UFRGS-PV-1346-T-S2) | 469.6 |  |
| *Irajatherium hernandezi* (UFRGS-PV-1348-T-S1) | 86.3 | 86.3 |
| *Oligokyphus* sp*.* (UMZC T.1236a) | 116.3 | 106.4 |
| *Oligokyphus* sp. (UMZC T.1236b) | 89.0 |  |
| *Oligokyphus* sp. (UMZC T.1236c) | 130.0 |  |
| *Oligokyphus* sp. (UMZC T.1236d) | 90.3 |  |
| *Morganucodon watsoni* (UMZC 2017.4.1) | 30.2 | 38.9 |
| *Morganucodon watsoni* (UMZC 2017.4.2) | 47.5 |  |

**S9. References**

**Altmann J., Schoeller D., Altmann S.A., Muruthi P. and Sapolsky R.M. 1993.** Body size and fatness of free-living baboons reflect food availability and activity levels. *American Journal of Primatology* 30: 149-61.

**Benjamini Y. and Hochberg Y. 1995.** Controlling the false discovery rate: a practical and powerful approach to multiple testing. *Journal of the Royal Statistical Society, Series B, Statistical Methodology* 57: 289**-**300.

**Brown R.P. and Yang Z. 2010.** Bayesian dating of shallow phylogenies with a relaxed clock. *Systematic* Biology 59: 119-131.

**Curran‐Everett D. 2000.** Multiple comparisons: philosophies and illustrations. *American Journal of Physiology. Regulatory, Integrative and Comparative Physiology* 279: 1**-**8.

**Cott H.B. 1961.** Scientific results of an inquiry into the ecology and economic status of the Nile crocodile (*Crocodylus niloticus*) in Uganda and northern Rhodesia. *Transactions of the Zoological Society of London* 29: 211**–**256.

**Davies P. and Lister A.M. 2001.** *Paleoloxodon cypriotes*, the dwarf elephant of Cyprus: size and scaling comparisons with *P. falconeri* (Sicily-Malta) and mainland *P. antiquus*. *1st International Congress, The World of Elephants, Rome*, pp. 479-480.

**Dirks W., Anemone R.L., Holroyd P.A., Reid D.J. and Walton P. 2009.** Phylogeny, life history and the timing of molar crown formation in two archaic ungulates, *Meniscotherium* and *Phenacodus* (Mammalia,’Condylarthra’). In Koppe T., Meyer G. and Alt K.W. (eds.) *Comparative Dental Morphology. Frontiers of Oral Biology.* Karger, Basel. 13: 3-8.

**Dirks W., Bromage T.G. and Agenbroad L.D. 2012.** The duration and rate of molar plate formation in Palaeoloxodon cypriotes and *Mammuthus columbi* from dental histology. *Quaternary International* 255: 79-85.

**Fitch W.T. 2000.** Skull dimensions in relation to body size in non-human mammals: the causal basis for acoustic allometry. *Zoology* 103: 40-58.

**Gaetano L.C. and Abdala F. 2015.** The stapes of gomphodont cynodonts: insights into the middle ear structure of non-mammaliaform cynodonts. *PLoS ONE* 10: e0131174.

**Gingerich P.D. 2003.** Mammalian responses to climate change at the Paleocene-Eocene boundary: Polecat Bench record in the northern Bighorn Basin, Wyoming. In: Wing S.L., Gingerich P.D., Schmitz B. and Thomas E. (eds.) *Causes and Consequences of Globally Warm Climates in the Early Paleogene.* Geological Society of America, Special Paper 369: 463-478.

**Halliday T.J.D., Upchurch P. and Goswami A. 2017**. Resolving the relationships of Paleocene placental mammals. *Biological Reviews* 92: 521-550.

**Hedges S.B., Marin J., Suleski M., Paymer M. and Kumar S. 2015**. Tree of Life Reveals Clock-Like Speciation and Diversification. *Molecular and Biological Evolution* 32: 835-845.

**Hurlburt G.R. 1996.** *Relative brain size in recent and fossil amniotes: determination and interpretation*. Ph.D. dissertation, University of Toronto, Toronto, Ontario, Canada.

**Jerison H.J. 1973.** *Evolution of the Brain and Intelligence*. Academic Press, New York.

**Jordana X. and Köhler M. 2011.** Enamel microstructure in the fossil bovid *Myotragus balearicus* (Majorca, Spain): implications for lifehistory evolution of dwarf mammals in insular ecosystems. *Palaeogeography Palaeoclimatology Palaeoecology* 300: 59-66.

**Kierdorf H., Kierdorf U., Frölich K. and Witzel C. 2013.** Lines of evidence – incremental markings in molar enamel of Soay sheep as revealed by a fluorochrome labeling and backscattered electron imaging study. *PLoS ONE* 8: e74597.

**Kierdorf H., Breuer F., Richards A. and Kierdorf U. 2014.** Characterization of enamel incremental markings and crown growth parameters in minipig molars. *The Anatomical Record* 297:1935-1949.

**Kieser J.A., Tkatchenko T., Dean M.C., Jones M.E.H., Duncan W. and Nelson, N.J. 2009.** Microstructure of dental hard tissues and bone in the tuatara dentary, *Sphenodon punctatus* (Diapsida: Lepidosauria: Rhynchocephalia). In Koppe T., Meyer G. and Alt K.W. (eds.) *Comparative Dental Morphology*. *Frontiers of Oral Biology*. Karger, Basel. 13: 80-85.

**Kijas J.W., Lenstra J.A., Ben Hayes B., Boitard S, Porto Neto L.R., San Cristobal M. et al. 2010.** Genome-wide analysis of the world’s sheep breeds reveals high levels of historic mixture and strong recent selection. *PLOS Biology* 10: e1001258.

**Koyabu D., Werneberg I., Morimoto N., Zollikofer C.P.E., Forasiepi A.M. 2014.** Mammalian skull heterochrony reveals modular evolution and a link between cranial development and brain size. *Nature Communications* 5:3625**.**

**Kühne W.G. 1956.** *The Liassic therapsid* Oligokyphus. Trustees of the British Museum, London.

**Lalueza-Fox C., Shapiro B., Bover P., Alcover J.A. and Bertranpetit J. 2002.** Molecular phylogeny and evolution of the extinct bovid *Myotragus balearicus*. *Molecular phylogenetic and Evolution* 25: 501-510.

**Luo Z.-X., Crompton A.W. and Sun A.-L. 2001.** A new mammal from the Early Jurassic and evolution of mammalian characteristics. *Science* 292: 1535-1540.

**de Magalhaes J.P. and Costa J. 2009.** A database of vertebrate longevity records and their relation to other life-history traits. *Journal of Evolutionary Biology* 22: 1770**-**1774.

**Meredith R.W., Janečka J.E., Gatesy J., Ryder O.A., Fisher C.A., Teeling E.C. et al. 2011.** Impacts of the Cretaceous Terrestrial Revolution and KPg Extinction on Mammal Diversification. *Science* 334: 521-524.

**Perelman P., Johnson W.E., Roos C., Seuánez H.N., Horvath J.E., Moreira M.A.M. et al. 2011.** A molecular phylogeny of living primates. *PloS Genetics* 7: e1001342.

**Poulakakis N., Parmakelis A., Lymberakis P., Mylonas M., Zouros E., Reese D.S., Glaberman S. and Caccone A. 2006.** Ancient DNA forces reconsideration of evolutionary history of Mediterranean pygmy elephantids. *Biology Letters* 2: 451–454.

**Milner J.M., Albon S.D., Illius A.W., Pemberton J.M. and Clutton-Brock T.H. 1999.** Repeated selection of morphometric traits in the Soay sheep on St Kilda. *Journal of Animal Ecology* 68: 472-488.

**Nunoya T., Shibuya K., Saitoh T., Yazawa H., Nakamura K., Baba Y. and Hirai T. 2007.** Use of miniature pig for biomedical research, with reference to toxicologic studies. *Journal of Toxicologic Pathology* 20: 125-132.

**Oliveira T.V., Martinelli A.G. and Soares M.B. 2011.** New information about *Irajatherium hernandezi* Martinelli, Bonaparte, Schultz & Rubert 2005 (Eucynodontia, Tritheledontidae) from the upper triassic (Caturrita Formation, Paraná Basin) of Brazil. *Paläontologische Zeitung* **85**:67–82.

**Orme D., Freckleton R., Thomas G, Petzoldt T., Fritz S., Isaac N. and Pearse W. 2013.** caper: Comparative Analyses of Phylogenetics and Evolution in R. R package version 0.5.2. https://CRAN.R-project.org/package=caper.

**Paleobiology Database.** Estimated body mass of *Borealosuchus wilsoni* recorded in the Paleobiology Database, Fossilworks. http://fossilworks.org. Accessed 12/11/2015.

**Quiroga J.C. 1980.** The brain of the mammal-like reptile *Probainognathus jenseni* (Therapsida, Cynodontia). A correlative paleoneurological approach to the neocortex at the reptile-mammal transition. *Journal für Hirnforschung* 21: 299-326.

**Revell L.J. 2009.** Size-correction and principal components for interspecific comparative studies. *Evolution* 63: 3258–3268

**Rowe T., Macrini T.E., and Luo Z.-X. 2011.** Fossil evidence on origin of the mammalian brain. *Science* 332: 955-957.

**Ruta M., Botha-Brink J., Mitchell S.A., and Benton M.J. 2013.** The radiation of cynodonts and the ground plan of mammalian morphological diversity. *Proceedings of the Royal Society B* 280: 20131865.

**Sehic A. Nirvani M. and Risnes S. 2013.** Incremental lines in mouse molar enamel. *Archives of Oral Biology* 58: 1443-1449.

**Shellis R.P. 1998.** Utilization of periodic markings in enamel to obtain information on tooth growth. *Journal of Human Evolution* 35: 387-400.

**Shipman P. 1992.** Body size and broken bones: preliminary interpretations of proboscidean remains. In: Fox J.W., Smith C.B. and Wilkins K.T. (eds.), *Proboscidean and Paleoindian Interactions*. Baylor University Press, Waco, pp. 75-98.

**Skinner J. and Smithers R. 1990.** *The Mammals of the Southern African Subregion*, 2nd edn. University of Pretoria, South Africa.

**Sokal R.R. and Rohlf F.J. 2012.** *Biometry*, 4^th^ edn. W. H. Freeman and Company, New York, NY.

**Tabachnick B.G. and Fidell L.S. 2007.** *Using Multivariate Statistics*, 5^th^ edn. Pearson Education Inc., Boston.

**Thewissen J.G.M. 1990.** Evolution of Paleocene and Eocene Phenacodontidae (Mammalia, Condylarthra). *University of Michigan Papers on Paleontology* 29: 1-107.

**Williamson T.E. and Lucas S.G. 1992.** *Meniscotherium* (Mammalia, ‘Condylarthra’) from the Paleocene-Eocene of Western North America. *New Mexico Museum of Natural History and Science Bulletin* 1:1-75.
